# First Total Synthesis of Pestasulfamides A and B Through Iminoketene Dimerization of Anthranilic Acid in One-Pot Manner

**DOI:** 10.3390/molecules31010047

**Published:** 2025-12-22

**Authors:** Yuito Kobori, Takumi Abe

**Affiliations:** Graduate School of Medicine, Dentistry and Pharmaceutical Sciences, Okayama University, Okayama City 700-8530, Japan

**Keywords:** total synthesis, natural products, phenylbenzene sulfonamides, dibenzodiazocin-2,6-dione

## Abstract

Pestasulfamides A and B are phenylbenzene-sulfonamides with an eight-membered dilactam, produced by mangrove endophytic fungus *Pestalotiopsis* sp. HNY36-1D. In bioassay, pestasulfamide A (**1**) exhibited potent anti-acetylcholine esterase (AChE) activities with an IC50 value of 11.94 μM, offering new pharmacophores with relevance to anti-Alzheimer’s disease drug discovery. Although the dimerization reaction of anthranilic acid derivatives forges an dibenzodiazocin-2,6-dione framework, the application of the dimerization to total synthesis of pestasulfamides A (**1**) and B (**2**) has not yet been realized. Herein, the first total synthesis of pestasulfamides A and B was achieved through one-pot protocol. The key step features a sulfonylation-induced iminoketene dimerization of anthranilic acid in a pyridine/THF system.

## 1. Introduction

Primary sulfonamides are known as privileged drug pharmacophore and are widely used as drugs [1,2,3,4]. With superior physical property, metabolic stability, and easiness to synthesis, primary and secondary sulfonamides have attracted much attention from synthetic and medicinal chemists. Among the sulfonamide family, a tertiary sulfonamide has not been pushed to the forefront of the drug design, probably due to the lack of acidic hydrogen atom at the nitrogen, which can act as a hydrogen bonding donor. Unlike that trend, recent studies revealed that some tertiary sulfonamides can play a novel pharmacophore, probably due to their good binding properties against target enzymes and/or receptors such as liver X receptor antagonists (LXRs) [5], butylcholinesterase inhibitors for the treatment of Alzheimer’s disease [6], antiproliferative activities [7], and α-carbonic anhydrase of *Helicobacter pylori* (HpαCA) inhibitors [8]. On the other hand, natural products containing sulfonamide units are relatively rare, although drug-like compounds containing sulfonamides are well-developed [9].

**Figure 1:**
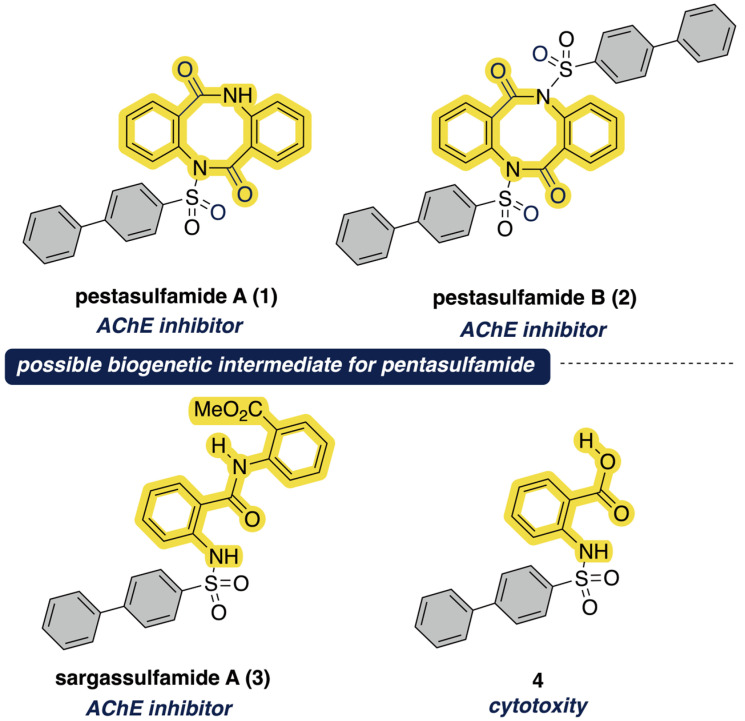
Structures of phenylbenzenesulfonyl-substituted natural products.

In this context, phenylbenzenesulfonyl amide-type natural products, pestasulfamides A (**1**) and B (**2**), were isolated in 2023 from mangrove endophytic fungus *Pestalotiopsis* sp. HNY36-1D by the Sun and She group (Figure 1) [10]. The structures of **1** and **2** were determined by 1D and 2D NMR, HR-ESIMS, IR, and a single crystal X-ray diffraction for **2**. Structurally, pestasulfamides A (**1**) and B (**2**) are rare dibenzodiazocines that differ in their number of phenylbenzenesulfonyl amide groups. In bioassay, pestasulfamide A (**1**) exhibited potent anti-acetylcholine esterase (AChE) activities with IC50 value 11.94 μM, while pestasulfamides B (**2**) did not show AChE inhibitory activity. Authors conclude that the origin of AChE inhibitory activity comes from the closed loop of cyclic dibenzodiazocines, which is caused by hydrogen bonding of nitrogen atom of **1**. In this isolation report, known sargassulfamide A (**3**) were also isolated as acyclic analog with two cyclic analogs **1** and **2**, which suggests that sargassulfamide A (**3**) or known phenylbenzenesulfonyl amide natural product (**4**) may be a bioprecursor of pestasulfamides A (**1**) and B (**2**) through stepwise amidation/cyclization [11,12]. Among sulfonamide natural products [13,14], phenylbenzenesulfonyl amides are quite rare [15,16].

**Scheme 1:**
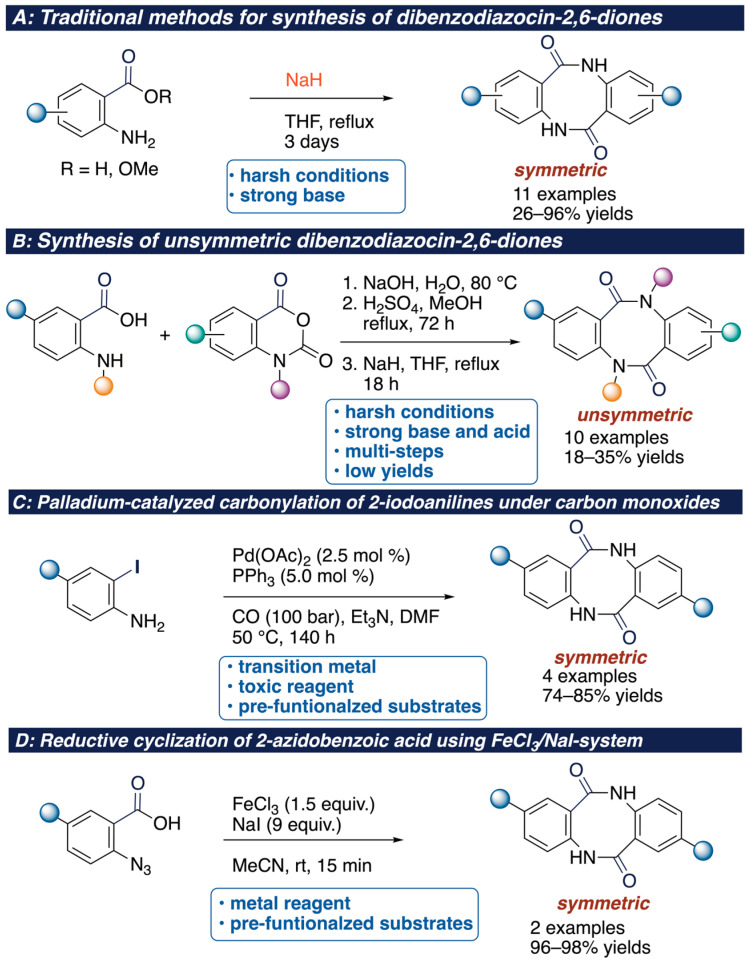
State-of-the-art of synthesis of dibenzodiazocin-2,6-diones.

Dibenzodiazocin-2,6-diones have drawn attention from organic chemists due to their cyclic diamide flexible eight-membered structure [17], intriguing planner chirality [18,19,20], and chemosensitivities [21]. Traditional methods for the synthesis of dibenzodiazocin-2,6-diones include a base-promoted dimerization of anthranilic acid derivatives (Scheme 1A) [22,23,24,25]. However, such approaches face certain limitations, including reflux reaction conditions, use of the strong base such as NaH, and being restricted to anthranilic acid only. Alternatively, condensation of anthranilic acid with isatoic anhydrides could provide unsymmetric dibenzodiazocin-2,6-diones, bearing a variety of substituents, still albeit low yields (18–35% yields, 11 examples) (Scheme 1B) [26]. Although palladium-catalyzed carbonylation of 2-iodoanilines is also regarded as an efficient strategy toward dibenzodiazocin-2,6-diones, it requires both expensive transition metal and highly toxic carbon monoxide (Scheme 1C) [27]. Kamal’s group reported the reductive cyclization of 2-azidobenzoic acid using excess amount of FeCl_3_ to afford dibenzodiazocin-2,6-diones (Scheme 1D) [28]. However, these methods are complicated by the obliged preparation of the functionalized iodoanilines and 2-azidobenzoic acids. And the use of transition metals results in limitations in the functional group tolerance of the starting materials.

Moreover, there have been many synthetic routes to dibenzodiazocine derivatives developed. The Tang and Li group developed the base-promoted protocol for the construction of an eight-membered ring using o-aminobenzonitrile or o-phenylenediamine/phthalonitrile derivatives [29]. Wan and co-workers reported an acid-medated dimerization of isocyanates intermediates generated from benzoyl azides [30]. Authors hypothesized that the mechanism involves intermolecular [2 + 2] cyclization between isocyanates and ketone moieties. Similar casade reactions involving unique mechanism using specific substrates or reagents have been reported. For instance, Scandium Pentafluorobenzoate-catalyzed cascade reaction of *o*-aminobenzaldehydes with primary amines affords an aminal-type eight-membered ring [31]. Grignard reagents with o-aminobenzonitriles generates dianion intermediates, resulting in the rapid dimerization and production of an aminal-type eight-membered ring [32]. Combination use of gold catalyst [(JonPhosAuNCMe)SbF_6_] and β-(2-aminophenyl)-α,β-ynones affords dibenzo [1,5]diazocines through the selective *8-exo-dig* intramolecular hydroamination of alynes [33]. Although the dimerization reaction of anthranilic acid derivatives forges an dibenzodiazocin-2,6-dione, application of the dimerization to total synthesis of pestasulfamides A (**1**) and B (**2**) has not yet been realized [34].

**Scheme 2:**
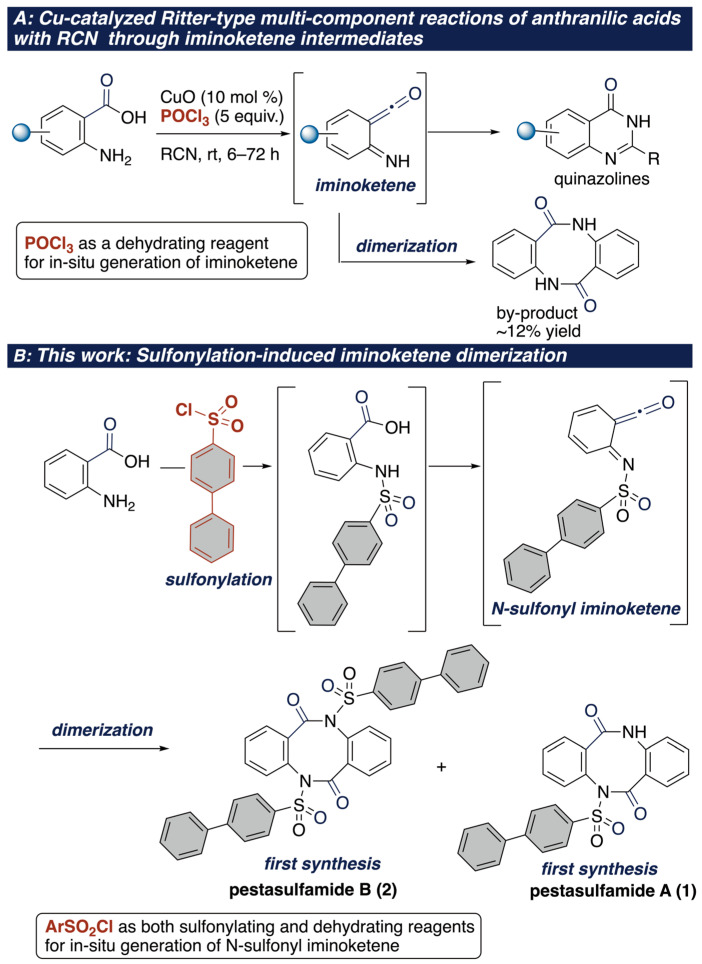
(**A**) Our developed Ritter-type reaction of anthranilic acids with RCN using POCl_3_ as a dehydrating reagent in the presence of copper catalyst; (**B**) This work (sulfonylation-induced iminoketene dimerization).

In our continuation of the impressive efforts to access heterocycles through aminative coupling reactions [35,36,37], we developed the one-pot synthesis of *trans*-chloroamidines through a copper-catalyzed Ritter-type multicomponent reaction of unactivated alkenes with various nitriles, using dichloramine-T as an electrophile [38]. As an extension of the Ritter-type reaction, we also developed a Ritter-type reaction of anthranilic acids with various nitriles to afford quinazoline, assisted by POCl_3_ in the presence of copper catalyst (Scheme 2A) [39]. During this study, we encountered an unexpected dibenzodiazocin-2,6-dione, albeit low yield, through an iminoketene dimerization. Prompted by this unexpected observation, we envisioned that such an iminoketene dimerization strategy would be applicable to access pestasulfamides A (**1**) and B (**2**) if a suitable acid promoter is discovered. Herein, we report the first total synthesis of pestasulfamides A (**1**) and B (**2**) utilizing the dimerization of anthranilic acid derivatives (Scheme 2B). Notably, this represents the first example of utilizing the iminoketene dimerization of anthranilic acids in natural product synthesis.

## 2. Results and Discussion

**Scheme 3:**
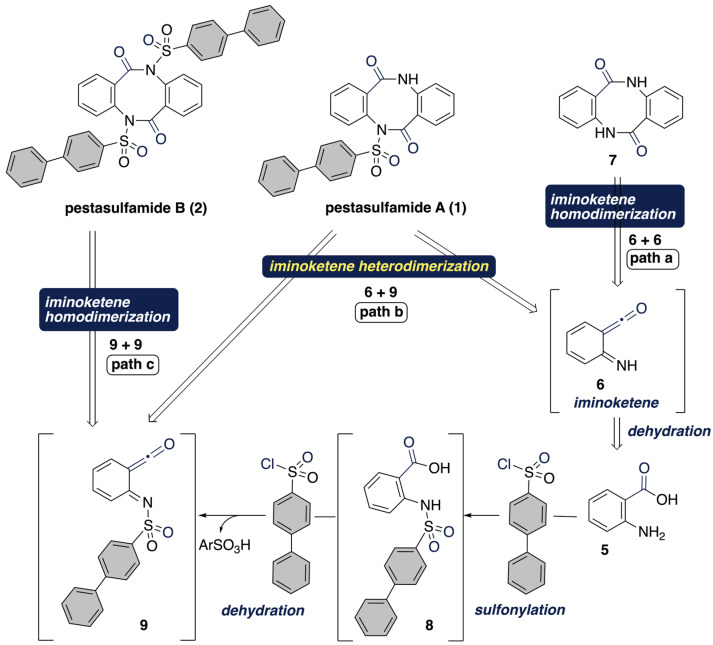
Retrosynthetic analysis of synthesis of pestasulfamides A (**1**) and B (**2**).

Building upon our previous observation in the Ritter-type reaction using anthranilic acid (**5**) with POCl_3_, we envisioned the production of *N*,*N*’-unsubstituted benzodiazocine **7** and pestasulfamides A (**1**) and B (**2**) by acid-promoted divergent iminokete dimerization through *path a* (NH-iminoketene **6**), *path b* (iminoketenes **6** and **9**), and *path c* (N-substituted iminoketene **9**), respectively (Scheme 3). The assembly of anthranilic acid (**5**) and 4-phenylbenzenesulfonyl chloride would lead to N-sulfonyl iminoketene **9** through sulfonamide **8**, simultaneously. Then, the dimerization of N-sulfonyl iminoketene **9** occurs to produce pestasulfamide B (**2**) (path c). The timing of in situ sulfonylation of amine moieties plays an important role in our envisioned transformations.

To assess the feasibility of this synthetic blueprint, we tested our synthetic blueprint with anthranilic acid (**5**) and 4-phenylbenzenesulfonyl chloride as a promoter for generation of iminoketene intermediate in pyridine at room temperature (Table 1). An equimolar amount of 4-phenylbenzenesulfonyl chloride relative to anthranilic acid led to low conversion, affording products **1** and **2** in 21% and 8% (dimer yields), respectively (run 1). Contrary to expectations, benzodiazocine **7** was not detected from the reaction mixture, presumably due to less efficient dimerization of unsubstituted iminoketene **6**. This observation suggests that the N-sulfonylation of anthranilic acid plays an important role to promote the expected dimerization. Thus, we next investigated the 4-phenylbenzenesulfonyl chloride-to-substrate **5** ratio. Further investigations revealed that the amount of sulfonyl chloride played an important role in operating our transformation. The use of 1.5 equiv of 4-phenylbenzenesulfonyl chloride afforded products **1** and **2** in 33% and 16%, respectively (run 2). The use of 2 equiv of 4-phenylbenzenesulfonyl chloride was most effective, resulting in the isolation of products **1** and **2** in 47% and 37%, respectively (run 3). Neither increasing 4-phenylbenzenesulfonyl chloride to 2.5 equiv nor increasing it to 3.0 equiv slightly diminished the product yields, presumably due to the solubility issue (runs 4 and 5). Next, we investigated the impact of temperature on the reaction outcome (runs 6–8). The drop of temperature (0 °C) led to reduced yields (run 6), whereas increasing temperature has no discernible impact on the product yields (runs 7 and 8). Notably, the reaction rate was not affected by reaction temperature. Solvents have a great impact on the reaction outcome. No products **1** and **2** were afforded by using other solvents such as Et_3_N, AcOEt, THF, and DMSO (runs 9–12). These results revealed that pyridine is required to operate our transformation. Of the co-solvents screened (runs 13–15), the pyridine/THF system (1/1, *v*/*v*) proved slight improved yields (run 13). Non-protic polar solvents such as DMF and DMSO showed lower yields, probably due to the stabilization of cationic intermediate by these solvents.

In isolation paper by Sun and She group, 2D NMR experiments of pestasulfamides A (**1**) and B (**2**) were insufficient due to the limit quantity of pestasulfamides A (**1**) and B (**2**) (A: 1.9 mg, B: 2.4 mg), while the structure of pestasulfamide B (**2**) was confirmed by X-ray analysis. With sufficient amount of compound with the purity acceptable for the NMR studies in hand, we conducted 2D NMR experiments. The spectroscopic data obtained for the synthetic sample of products **1** and **2** were identical to those reported for isolated pestasulfamides A (**1**) and B (**2**) (Table 2 and Table 3). By the 2D NMR studies, full NMR assignment of pestasulfamides A (**1**) and B (**2**) could be presented (Supplementary Materials). Moreover, the 2D NMR studies suggests some misassignment of natural products **1** and **2** at ^13^C-NMR, probably due to insufficient amounts of isolated products and insolubilities of these compounds.

**Scheme 4:**
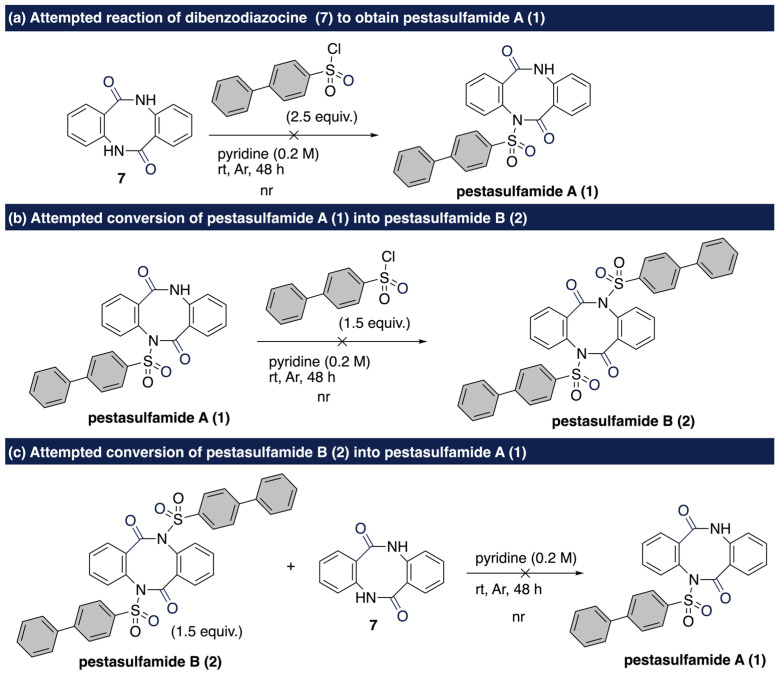
Control experiments to elucidate the reaction mechanism.

We then turned our attention to the timing of introduction of sulfonyl group. To gain further insight into the timing of the introduction of 4-phenylbenzenesulfonyl group on the dibenzodiazocine core, several additional experiments were conducted (Scheme 4). First, dibenzodiazocine **7** was subjected to the standard reaction conditions for 48 h, which resulted in no reaction (Scheme 4a). Furthermore, the reaction using possible intermediate **1** for pestasulfamide B (**2**) was conducted (Scheme 4b). To our surprise, no product was detected, thereby supporting the concerted dimerization mechanism. On these observations, the late-stage transfer reaction of the sulfonyl group using **2** and **7** was performed (Scheme 4c). In that case, we could not detect the desired product **1**, which would be produced by transfer of the sulfonyl group from disulfonylated compound **2**. These results render the stepwise cyclization/gradual sulfonamidation pathway unfeasible.

**Scheme 5:**
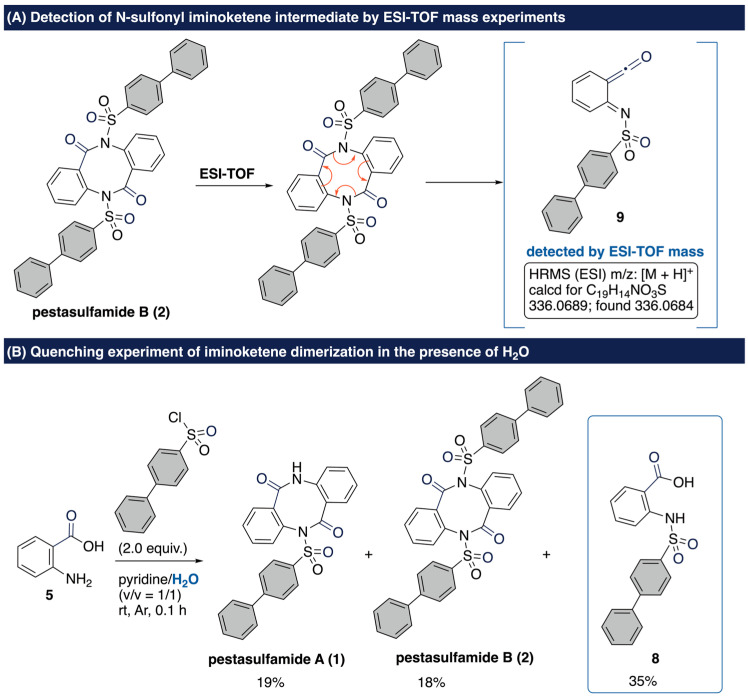
Confirmation of iminoketene pathway.

We conducted some mechanistic experiments to confirm the iminoketene dimerization pathway. First, the retro mass-spectral synthesis [40] afforded iminoketene fragment ion **9** through the retro-dimerization-type cleavage of pestasulfamide B (**2**) (Scheme 5A). Detection of the iminoketene fragment ion **9** suggests that the dimerization process involves the generation of iminoketene species. Next, the quenching experiments in the presence of H_2_O afforded no dimerized product **8** in 35% yield along with pestasulfamides (Scheme 5B). The result of the quenching experiment strongly supports our hypothesis that N-sulfonylanthranilic acid **8**, which gives rise to the generation of N-sulfonyl iminoketene intermediate **9**, is indeed involved in our transformation.

## 3. Materials and Methods

### 3.1. General Information

Column chromatography was carried out using silica gel (WAKO Gel 75–150 mesh, FUJIFILM Wako Pure Chemical Co., Osaka, Japan). Preparative tin-layer chromatography was performed with silica gel plates (0.25 mm, silica gel, 60F_254_, Merk & Co., Inc., Keniworth, NJ, USA). Melting points (mp) were recorded with a Yamato melting point apparatus model MP-21 (Yamato Scientific Co., Ltd., Tokyo, Japan) and are uncorrected. IR spectra were measured with a HORIBA Fourier transform infrared spectrometer FT-720 (HORIBA Ltd., Kyoto, Japan) and absorbance frequencies are reported in reciprocal centimeters (cm^−1^). NMR experiments were performed with JEOL JNM-ECZ600R (JEOL Ltd., Tokyo, Japan) (^1^H NMR: 600 MHz, ^13^C NMR: 151 MHz) spectrometer and Varian NMR system 600 (^1^H NMR:600 MHz, ^13^C NMR: 151 MHz) spectrometer (Agilent Technologies Japan, Ltd., Tokyo, Japan). Chemical shifts are expressed in *δ* [parts per million (ppm)] values and coupling constants are expressed in hertz (Hz). ^1^H NMR spectra were referenced to a solvent signal (CDCl_3_: 7.26 ppm, DMSO-*d_6_*: 2.50 ppm). ^13^C NMR spectra were referenced to a solvent signal (CDCl_3_: 77.1 ppm, DMSO-*d_6_*: 39.5). Signal multiplicities are abbreviated as follows: singlet (s), doublet (d), triplet (t), multiplet (m), doublet of doublets (dd), triplet of doublets (td), and triplet of triplets (tt). High-resolution MS spectra were recorded with a Brucker microTOF mass spectrometer (ESI-TOF-MS) (Bruker Co., Billerica, MA, USA). Reactions were monitored by thin layer chromatography (TLC) carried out on a silica gel plate (60F_254_) and visualized under UV illumination at 254 or 365 nm, depending on the compounds. All substrates were used as received from commercial suppliers [Sigma-Aldrich (Merk & Co., Inc., Keniworth, NJ, USA), Kanto Chemical (Kanto Chemical Co. Inc., Tokyo, Japan) TCI (Tokyo Chemical Industry Co., Ltd., Tokyo, Japan), Wako (FUJIFILM Wako Pure Chemical Co., Osaka, Japan), and Nacalai Tesque (Nacalai Tesque, Inc., Kyoto, Japan)] and all reagents were weighed and handled in air at room temperature.

### 3.2. Experimental Procedures and Characterization Data for the Synthetic Products

#### 3.2.1. Synthesis of Pestasulfamide A (**1**) and Pestasulfamide B (**2**)

[Typical procedure for run 3, Table 1]

A solution of **5** (137 mg, 1.0 mmol) in pyridine (5.0 mL, 0.2 M) was added to 4-biphenylsulfonyl chloride (505 mg, 2.0 equiv., 2.0 mmol) under Ar atmosphere. The mixture was stirred at room temperature for 0.1 h. After the addition of 0.1 M HCl (20 mL), the whole was extracted with AcOEt (3 × 20 mL). The combined organic layer was extracted with sat. NaHCO_3_ (20 mL), washed with brine (20 mL), dried over Na_2_SO_4_, filtered, and concentrated *in vacuo*. The residue was purified by silica gel column chromatography using hexane/AcOEt [3/1 (*v*/*v*)] to give **1** (107 mg, 0.24 mmol, 47% dimer yield) as a white solid and **2** (125 mg, 0.19 mmol, 37% dimer yield) as a white solid.

5-([1,1′-Biphenyl]-4-ylsulfonyl)dibenzo[*b*,*f*][1,5]diazocine-6,12(5*H*,11*H*)-dione (pestasulfamide A, **1**): Mp 273–275 °C dec.; IR (KBr) ν: 3539, 3093, 2979, 1711, 1653, 1564, 1498, 1282, 1093, 814 cm^−1^; ^1^H NMR (600 MHz, CDCl_3_) *δ* 12.38 (s, 1H), 8.24 (dd, *J* = 7.8, 1.2 Hz, 1H), 8.19 (dd, *J* = 7.8, 1.8 Hz, 1H), 7.89–7.93 (m, 3H), 7.78 (t, *J* = 9.0 Hz, 2H), 7.60 (td, *J* = 8.4, 1.2 Hz, 1H), 7.57 (d, *J* = 8.4 Hz, 2H), 7.49–7.51 (m, 3H), 7.42 (t, *J* = 7.8 Hz, 2H), 7.38 (tt, *J* = 9.9, 1.1 Hz, 1H), 7.16 (td, *J* = 8.7, 1.0 Hz, 1H); ^13^C{^1^H} NMR (151 MHz, CDCl_3_) *δ* 158.1, 157.2, 146.1, 145.0, 139.8, 139.1, 138.1, 137.3, 134.3, 129.9, 129.3, 129.1, 129.0, 128.7, 127.8, 127.3, 126.8, 123.5, 119.5, 116.7, 115.5; HRMS (ESI) *m*/*z*: [M+Na]^+^ calcd for C_26_H_18_N_2_O_4_SNa 477.0880; found 477.0881.

5,11-Bis([1,1′-biphenyl]-4-ylsulfonyl)dibenzo[*b*,*f*][1,5]diazocine-6,12(5*H*,11*H*)-dione (pestasulfamide B, **2**): Mp 214–216 °C, dec.; IR (KBr) *v*: 3032, 2970, 1732, 1564, 1481, 1450, 1367, 1244, 1173, 1080, 768 cm^−1^; ^1^H NMR (600 MHz, CDCl_3_) *δ* 8.11 (d, *J* = 8.4 Hz, 4H), 7.75 (d, *J* = 8.4 Hz, 4H), 7.61 (d, *J* = 7.2 Hz, 4H), 7.40–7.47 (m, 8H), 7.33–7.35 (m, 4H), 7.15–7.18 (m, 2H); ^13^C{^1^H} NMR (151 MHz, CDCl_3_) *δ* 165.6, 147.4, 139.2, 136.1, 134.3, 133.5, 132.1, 130.7, 130.3, 129.3, 129.1, 129.0, 128.8, 127.8, 127.6; HRMS (ESI) *m*/*z*: [M+Na]^+^ calcd for C_38_H_26_N_2_O_6_S_2_Na 693.1125; found 693.1129.

#### 3.2.2. Typical Procedure for Run 8, Table 1

A solution of **5** (137 mg, 1.0 mmol) in pyridine (5.0 mL, 0.2 M) was added to 4-biphenylsulfonyl chloride (505 mg, 2.0 equiv., 2.0 mmol) under Ar atmosphere. The mixture was stirred at reflux in an oil bath for 0.1 h. After the addition of 0.1 M HCl (20 mL), the whole was extracted with AcOEt (3 × 20 mL). The combined organic layer was extracted with sat. NaHCO_3_ (20 mL), washed with brine (20 mL), dried over Na_2_SO_4_, filtered, and concentrated *in vacuo*. The residue was purified by silica gel chromatography using hexane/AcOEt [3/1 (*v*/*v*)] to give **1** (114 mg, 0.25 mmol, 50% dimer yield) as a white solid and **2** (133 mg, 0.20 mmol, 40% dimer yield) as a white solid.

#### 3.2.3. Typical Procedure for Run 13, Table 1

A solution of **5** (137 mg, 1.0 mmol) in pyridine (2.5 mL) was added to 4-biphenylsulfonyl chloride (505 mg, 2.0 equiv., 2.0 mmol) under Ar atmosphere. Then, THF (2.5 mL) was added to the mixture and stirred at room temperature for 0.1 h. After the addition of 0.1 M HCl (20 mL), the whole was extracted with AcOEt (3 × 20 mL). The combined organic layer was extracted with sat. NaHCO_3_ (20 mL), washed with brine (20 mL), dried over Na_2_SO_4_, filtered, and concentrated *in vacuo*. The residue was purified by silica gel column chromatography using hexane/AcOEt [3/1 (*v*/*v*)] to give **1** (113 mg, 0.25 mmol, 50% dimer yield) as a white solid and **2** (136 mg, 0.20 mmol, 41% dimer yield) as a white solid.

#### 3.2.4. Typical Procedure for Run 14, Table 1

A solution of **5** (137 mg, 1.0 mmol) in pyridine (2.5 mL) was added to 4-biphenylsulfonyl chloride (505 mg, 2.0 equiv., 2.0 mmol) under Ar atmosphere. Then, DMSO (2.5 mL) was added to the mixture and stirred at room temperature for 0.1 h. After the addition of 0.1 M HCl (20 mL), the whole was extracted with AcOEt (3 × 20 mL). The combined organic layer was extracted with sat. NaHCO_3_ (20 mL), washed with brine (20 mL), dried over Na_2_SO_4_, filtered, and concentrated *in vacuo*. The residue was purified by silica gel column chromatography using hexane/AcOEt [3/1 (*v*/*v*)] to give **1** (53 mg, 0.12 mmol, 23% dimer yield) as a white solid and **2** (53 mg, 0.08 mmol, 16% dimer yield) as a white solid.

#### 3.2.5. Typical Procedure for Run 15, Table 1

A solution of **5** (137 mg, 1.0 mmol) in pyridine (2.5 mL) was added to 4-biphenylsulfonyl chloride (505 mg, 2.0 equiv., 2.0 mmol) under Ar atmosphere. Then, DMF (2.5 mL) was added to the mixture and stirred at room temperature for 0.1 h. After the addition of 0.1 M HCl (20 mL), the whole was extracted with AcOEt (3 × 20 mL). The combined organic layer was extracted with sat. NaHCO_3_ (20 mL), washed with brine (20 mL), dried over Na_2_SO_4_, filtered, and concentrated *in vacuo*. The residue was purified by silica gel column chromatography using hexane/AcOEt [3/1 (*v*/*v*)] to give **2** (49 mg, 0.07 mmol, 15% dimer yield) as a white solid.

### 3.3. Preparation of Dibenzo[b,f][1,5]Diazocine-6,12 (5H,11H)-Dione (**7**) [41]

To the suspension NaH (1.6 g, 2.0 equiv., 40 mmol) in anhydrous THF (80 mL, 0.25 M), methyl 2-aminobenzoate (2.562 mL, 20 mmol) was added dropwise under Ar atmosphere. The mixture was stirred and heated to reflux for 3 days. The whole mixture was cooled to room temperature and then poured slowly into 0.1 M HCl with ice. After the ice melted, the precipitated product was collected by filtration and washed several times with H_2_O and AcOEt to yield **7** as a white solid (1.55 g, 6.5 mmol, 65% dimer yield). Mp 327–329 °C, dec.; IR (KBr) *v*: 3244, 3167, 1678, 1633, 835 cm^−1^; ^1^H NMR (600 MHz, CDCl_3_) *δ* 10.21 (s, 2H), 7.34 (td, *J* = 9.0, 1.4 Hz, 2H), 7.31 (dd, *J* = 7.5, 1.5 Hz, 2H), 7.23 (dt, J = 7.8, 0.6 Hz, 2H), 7.07 (d, *J* = 7.8 Hz, 2H); ^13^C{^1^H} NMR (151 MHz, CDCl_3_) *δ* 169.3, 134.8, 133.6, 130.6, 128.2, 127.3, 125.8; HRMS (ESI) *m*/*z*: [M+Na]^+^ calcd for C_14_H_10_N_2_O_2_Na 261.0635; found 261.0639.

### 3.4. Synthesis of 2-[([1,1′-Biphenyl]-4-Ylsulfonyl)Amino]Benzoic Acid (**8**) [42,43]

To a solution of **5** (137 mg, 1.0 mmol) in pyridine (2.5 mL), 4-biphenylsulfonyl chloride (505 mg, 2.0 equiv., 2.0 mmol) was added under Ar atmosphere. Then, H_2_O (2.5 mL) was added to the mixture and stirred at room temperature for 0.1 h. After the addition of 0.1 M HCl (20 mL), the whole was extracted with AcOEt (3 × 20 mL). The combined organic layer was extracted with sat. NaHCO_3_ (20 mL), washed with brine (20 mL), dried over Na_2_SO_4_, filtered, and concentrated in vacuo. The residue was purified by silica gel column chromatography using hexane/AcOEt [3/1 (*v*/*v*)] to give **1** (44 mg, 0.10 mmol, 19% dimer yield) as a white solid, **2** (61 mg, 0.09 mmol, 18% dimer yield) as a white solid, and **8** (125 mg, 0.35 mmol, 35% monomer yield) as a white solid.

2-([1,1′-biphenyl]-4-sulfonamide)benzoic acid (**8**): Mp 210–212 °C, dec.; IR (KBr) *v*: 3392, 3234, 3043, 1680, 1493, 1342, 1159, 761 cm^−1^; ^1^H NMR (600 MHz, DMSO-*d*_6_) *δ* 7.90 (d, *J* = 7.2 Hz, 1H), 7.84 (d, *J* = 8.4 Hz, 2H), 7.74 (d, *J* = 7.8 Hz, 2H), 7.63 (d, *J* = 7.2 Hz, 2H), 7.44 (q, *J* = 8.2 Hz, 3H), 7.39 (t, *J* = 7.2 Hz, 1H), 7.26 (t, *J* = 7.2 Hz, 1H), 6.85 (t, *J* = 7.5 Hz, 1H); ^13^C{^1^H} NMR (151 MHz, DMSO-*d*_6_) *δ* 170.9, 144.0, 143.3, 140.6, 138.9, 132.1, 131.7, 129.5, 128.8, 127.64, 127.56, 127.4, 122.3, 121.0, 117.4; HRMS (ESI) *m*/*z*: [M+Na]^+^ calcd for C_19_H_15_NO_4_SNa 376.0614; found 376.0616.

## 4. Conclusions

In summary, we have successfully developed a sulfonylation-induced dimerization of free anthranilic acid using an arylsulfonyl chloride as a sulfonylating and dehydrating reagent. This one-pot protocol provides a straightforward route to the natural products, pestasulfamides A (**1**) and B (**2**). In situ N-sulfonylation and dehydration sequences of anthranilic acid not only circumvent the conventional requirement for the harsh reaction conditions using strong bases, acids, and transition metals, but also reduce steps in the installation and/or removal of the protective group. To the best of our knowledge, our work showcases the first example of natural product synthesis through iminoketene dimerization of anthranilic acids. Moreover, divergent one-pot synthesis of both symmetric and unsymmetric dibenzodiazocines has been demonstrated. We believe our strategy can be applicable to a broad range of anthranilic acids and further applications are ongoing in our laboratory.

## Figures and Tables

**Table 1:** One-pot cascade reaction of anthranilic acid (**5**) towards pestasulfamides A (**1**) and B (**2**).

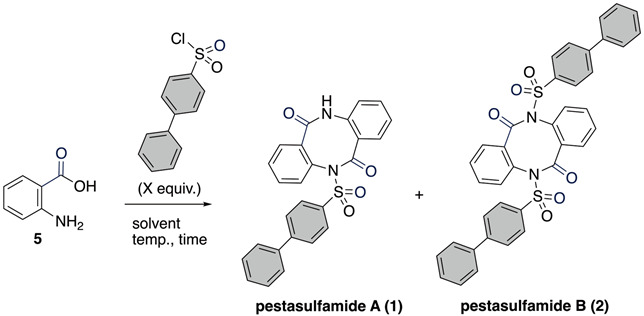						
Run *^a^*	PhBsCl (X Equiv.)	Solvent (0.2 M)	Temp. (°C)	Time (h)	Yield (%) of 1 *^b^*	Yield (%) of 2 *^b^*
1	1.0	pyridine	rt	0.1	21	8
2	1.5	pyridine	rt	0.1	33	16
3	2.0	pyridine	rt	0.1	47	37
4	2.5	pyridine	rt	0.1	40	39
5	3.0	pyridine	rt	0.1	35	20
6	2.0	pyridine	0	0.1	34	41
7	2.0	pyridine	60	0.1	46	45
8	2.0	pyridine	reflux	0.1	50	40
9	2.0	Et_3_N	rt	24	0	0
10	2.0	AcOEt	rt	24	0	0
11	2.0	THF	rt	24	0	0
12	2.0	DMSO	rt	24	0	0
13	2.0	pyridine/THF (1/1, *v*/*v*)	rt	0.1	50	41
14	2.0	pyridine/DMSO (1/1, *v*/*v*)	rt	0.1	23	16
15	2.0	pyridine/DMF (1/1, *v*/*v*)	rt	0.1	0	15

*^a^* Reaction conditions: Anthranilic acid (**5**) (1.0 mmol) and PhBsCl (X mmol, X equiv.) in solvent (5.0 mL, 0.2 M) under Ar. *^b^* Isolated yields.

**Table 2:** Comparison of ^1^H NMR (600 MHz) and ^13^C NMR (151 MHz) data in CDCl_3_ for synthetic sample **1** and ^1^H NMR (600 MHz) and ^13^C NMR (150 MHz) data for natural pestasulfamide A.

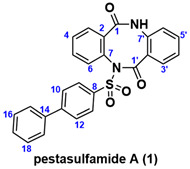				
Position	Synthetic 1 δ_H_ Mult (*J* in Hz) 600 MHz, CDCl_3_	Natural 1 δ_H_ Mult (*J* in Hz) 600 MHz, CDCl_3_	Synthetic 1 δc 151 MHz, CDCl_3_	Natural 1 δc 150 MHz, CDCl_3_
1			158.1	165.6
2			116.7	119.6
3	8.24, dd (1.2, 7.8)	7.93, dd (1.5, 8.1)	129.0	126.8
4	7.60, td (1.2, 8.4)	7.48, overlap	129.3	129.2
5	7.91, m	7.50, overlap	137.3	137.3
6	7.78, t (9.0)	7.62, m	126.8	116.8
7			145.0	139.9
8			138.1	143.7
9	7.91, m	7.71, overlap	127.8	128.7
10	7.57, d (8.7)	7.90, overlap	127.8	127.8
11			146.1	145.1
12	7.57, d (8.7)	7.90, overlap	127.8	127.8
13	7.91, m	7.71, overlap	127.8	128.7
14			139.1	146.2
15	7.50, m	7.57, overlap	127.3	128.7
16	7.42, t (7.8)	7.44, overlap	129.1	129.3
17	7.38, tt (1.1, 9.9)	7.38, overlap	128.7	127.4
18	7.42, t (7.8)	7.44, overlap	129.1	129.3
19	7.50, m	7.44, overlap	127.3	128.7
1′			157.2	163.4
2′			115.5	123.5
3′	8.19, dd (1.8, 7.8)	8.19, dd (1.5, 8.1)	129.9	129.9
4′	7.16, td (1.0, 8.7)	7.16, m	123.5	123.5
5′	7.50, m	7.52, overlap	134.3	134.3
6′	7.78, t (9.0)	8.25, dd (1.1, 7.9)	119.5	115.6
7′			139.8	138.2
1-NH	12.38, s	12.39, brs	---	---

**Table 3:** Comparison of ^1^H NMR (600 MHz) and ^13^C NMR (151 MHz) data in CDCl_3_ for synthesis **2** and ^1^H NMR (500 MHz) and ^13^C NMR (125 MHz) data for natural pestasulfamide B.

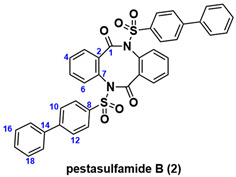				
Position	Synthetic 2 δ_H_ Mult (*J* in Hz) 600 MHz, CDCl_3_	Natural 2 δ_H_ Mult (*J* in Hz) 500 MHz, CDCl_3_	Synthetic 2 δc 151 MHz, CDCl_3_	Natural 2 δc 125 MHz, CDCl_3_
1			165.6	165.6
2			133.5	133.6
3	7.44, m	7.44, overlap	129.0	130.7
4	7.17, m	7.18, m	129.3	129.3
5	7.34, m	7.35, overlap	132.1	132.1
6	7.34, m	7.35, overlap	130.7	129.1
7			134.3	134.4
8			136.1	139.3
9	8.11, d (8.4)	7.77, d (7.9)	130.3	127.8
10	7.75, d (8.4)	8.13, d (7.9)	127.8	130.5
11			147.4	136.2
12	7.75, d (8.4)	8.13, d (7.9)	127.8	130.5
13	8.11, d (8.4)	7.77, d (7.9)	130.3	127.8
14			139.2	147.5
15	7.61, d (7.2)	7.62, d (7.2)	127.6	127.6
16	7.44, m	7.45, overlap	129.1	129.2
17	7.44, m	7.44, overlap	128.8	128.8
18	7.44, m	7.45, overlap	129.1	129.2
19	7.61, d (7.2)	7.62, d (7.2)	127.6	127.6

## Data Availability

The original contributions presented in this study are included in the article/Supplementary Material. Further inquiries can be directed to the corresponding author.

## Supplementary Materials

The following supporting information can be downloaded at: https://www.mdpi.com/article/10.3390/molecules31010047/s1. ^1^H- and ^13^C-NMR charts of all the compounds, and 2D-NMR charts of **1** and **2**.
