# Supplementary material for: First Total Synthesis of Pestasulfamides A and B Through Iminoketene Dimerization of Anthranilic Acid in One-Pot Manner
Source: Molecules. 2025 Dec 22;31(1):47. doi: 10.3390/molecules31010047 (PMC12786721; doi:10.3390/molecules31010047)
Supplement: Supplementary file 1 [file molecules-31-00047-s001.zip › molecules-4038238-supplementary.pdf]

First Total Synthesis of Pestasulfamides A and B Through Iminoketene Dimerization of Anthranilic Acid in One-Pot Manner

Yuito Kobori[1], and Takumi Abe*[1]

[1]Graduate School of Medicine, Dentistry and Pharmaceutical Sciences,
Okayama University, 1-1-1, Tsushima-naka, Kita-ku, Okayama 7008530, Japan
E-mail: t-abe@okayama-u.ac.jp

Supporting Information

1. Compound **1**: ^{1}H, ^{13}C NMR, and 2D NMR spectra ·· S2–S13

2. Compound **2**: ^{1}H, ^{13}C NMR, and 2D NMR spectra ·· S14–S23

3. Compound **7**: ^{1}H and ^{13}C NMR spectra ·· S24–S25

4. Compound **8**: ^{1}H and ^{13}C NMR spectra ·· S26–S27

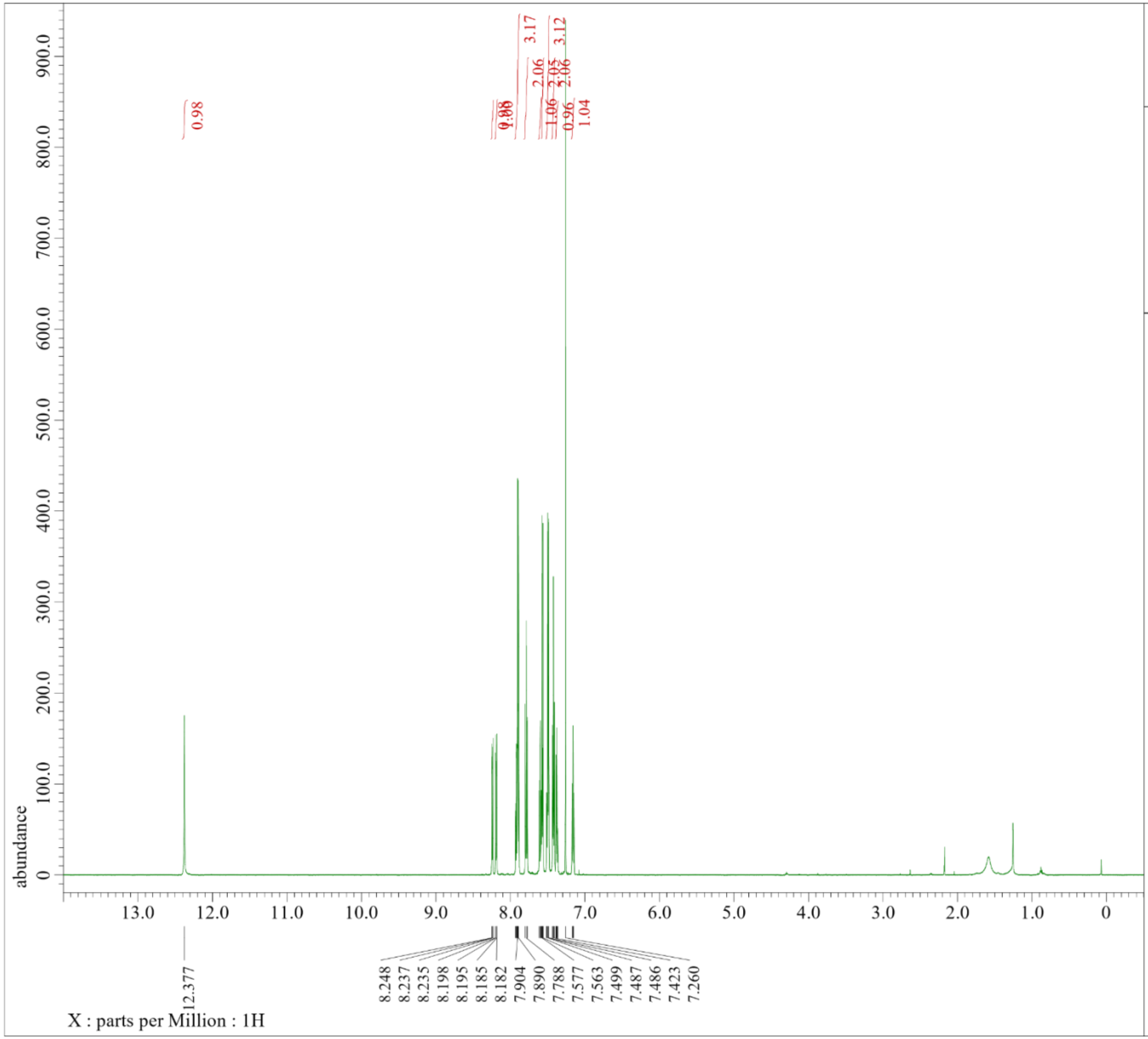


```
---- PROCESSING PARAMETERS ----
sexp( 0.2[Hz], 0.0[s] )
trapezoid( 0[%], 0[%], 80[%], 100[%] )
zerofill( 1, TRUE )
fft( 1, TRUE, TRUE )
machinephase
ppm
phase( -0.75, 29, 42.16437[%] )
reference( 7.25786[ppm], 7.26[ppm] )
base correct( Akima, 5, 0, FALSE, 3, None, FAL
phase( 0, 0, 50[%] )

以下に由来:: KY-4-146-4-1.jdf
```

```
Filename      = KY-4-146-4-2.jdf
Author        = Y.Kobori
Experiment    = s2pul
Solvent       = cdcl3
Revision_Time = 11-NOV-2025 18:38:16

Comment       = s2pul
Data_Format   = 1D REAL
Dim_Size      = 32768
X_Domain      = 1H
Dim_Title     = 1H
Dim_Units     = [ppm]
Dimensions    = X
Spectrometer  = VARIAN_UNITY_NMR

X_Domain      = 1H
X_Freq        = 599.76146205[MHz]
X_Freq_Flip   = FALSE
X_Offset      = 3.59855331[kHz]
X_Sweep       = 9.61538462[kHz]
Scans         = 8

Temp_Get      = 25[dC]
Ovj_P0        = -9.49612286[deg]
Ovj_P1        = 0[deg]
Ovj_Pp        = 100[%]
```

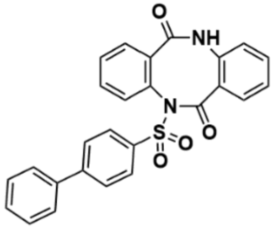

pestasulfamide A (1)

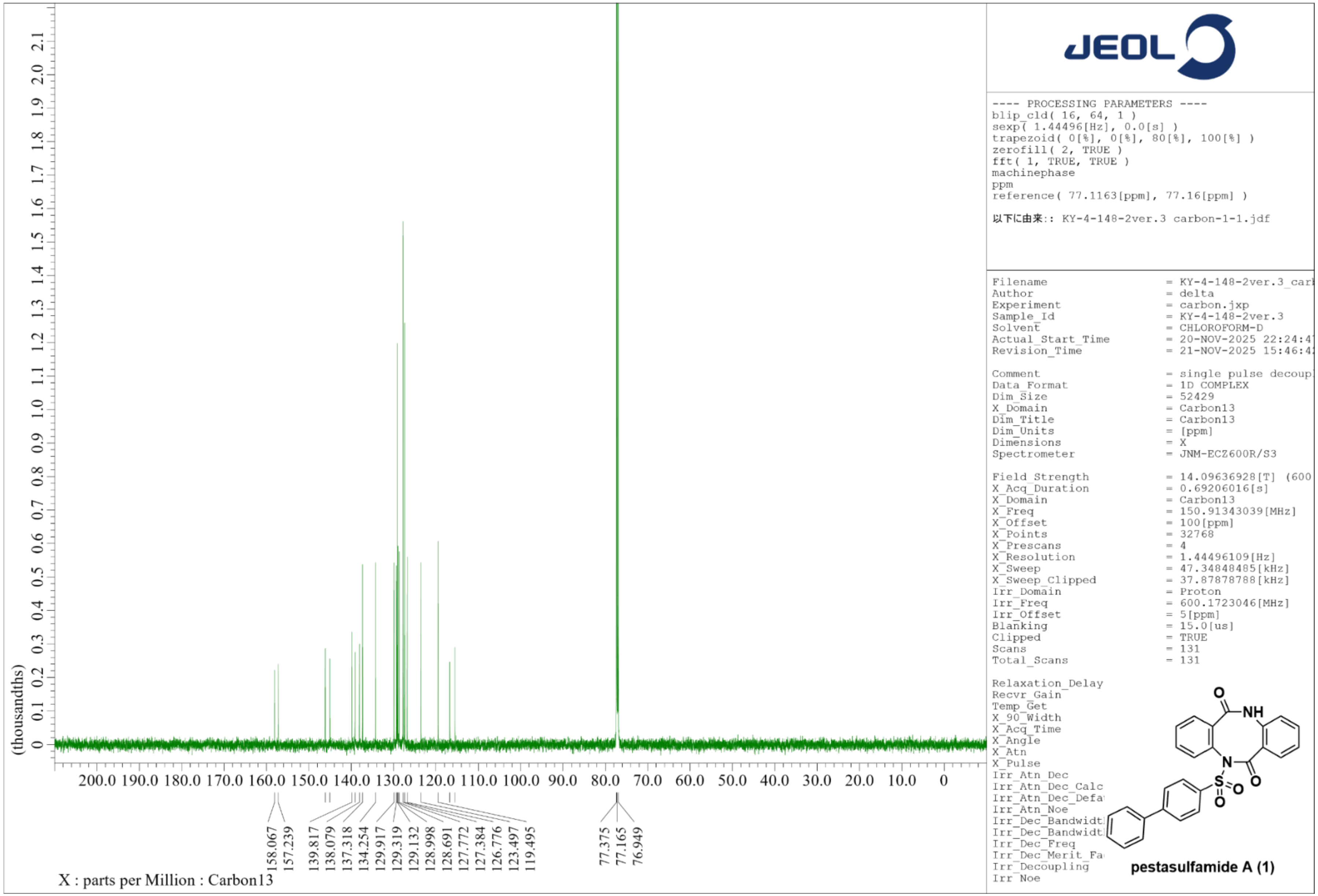
JEOL
---- PROCESSING PARAMETERS ----
blip_cld(16, 64, 1)
sexp(1.44496[Hz], 0.0[s])
trapezoid(0[%], 0[%], 80[%], 100[%])
zerofill(2, TRUE)
fft(1, TRUE, TRUE)
machinephase
ppm
reference(77.1163[ppm], 77.16[ppm])
以下に由来:: KY-4-148-2ver.3 carbon-1-1.jdf
Filename = KY-4-148-2ver.3_car
Author = delta
Experiment = carbon.jxp
Sample_Id = KY-4-148-2ver.3
Solvent = CHLOROFORM-D
Actual_Start_Time = 20-NOV-2025 22:24:4
Revision_Time = 21-NOV-2025 15:46:4
Comment = single pulse decoup
Data_Format = 1D COMPLEX
Dim_Size = 52429
X_Domain = Carbon13
Dim_Title = Carbon13
Dim_Units = [ppm]
Dimensions = X
Spectrometer = JNM-ECZ600R/S3
Field_Strength = 14.09636928[T] (600
X_Acq_Duration = 0.69206016[s]
X_Domain = Carbon13
X_Freq = 150.91343039[MHz]
X_Offset = 100[ppm]
X_Points = 32768
X_Prescans = 4
X_Resolution = 1.44496109[Hz]
X_Sweep = 47.34848485[kHz]
X_Sweep_Clipped = 37.87878788[kHz]
Irr_Domain = Proton
Irr_Freq = 600.1723046[MHz]
Irr_Offset = 5[ppm]
Blanking = 15.0[us]
Clipped = TRUE
Scans = 131
Total_Scans = 131
Relaxation_Delay
Recvr_Gain
Temp_Get
X_90_Width
X_Acq_Time
X_Angle
X_Atn
X_Pulse
Irr_Atn_Dec
Irr_Atn_Dec_Calc
Irr_Atn_Dec_Defa
Irr_Atn_Noe
Irr_Dec_Bandwidt
Irr_Dec_Bandwidt
Irr_Dec_Freq
Irr_Dec_Merit_Fa
Irr_Decoupling
Irr_Noe
O
NH
N
S
O
O
O
pestasulfamide A (1)
(thousandths)
0 0.1 0.2 0.3 0.4 0.5 0.6 0.7 0.8 0.9 1.0 1.1 1.2 1.3 1.4 1.5 1.6 1.7 1.8 1.9 2.0 2.1
200.0 190.0 180.0 170.0 160.0 150.0 140.0 130.0 120.0 110.0 100.0 90.0 80.0 70.0 60.0 50.0 40.0 30.0 20.0 10.0 0
158.067
157.239
139.817
138.079
137.318
134.254
129.917
129.319
129.132
128.998
128.691
127.772
127.384
126.776
123.497
119.495
77.375
77.165
76.949
X : parts per Million : Carbon13

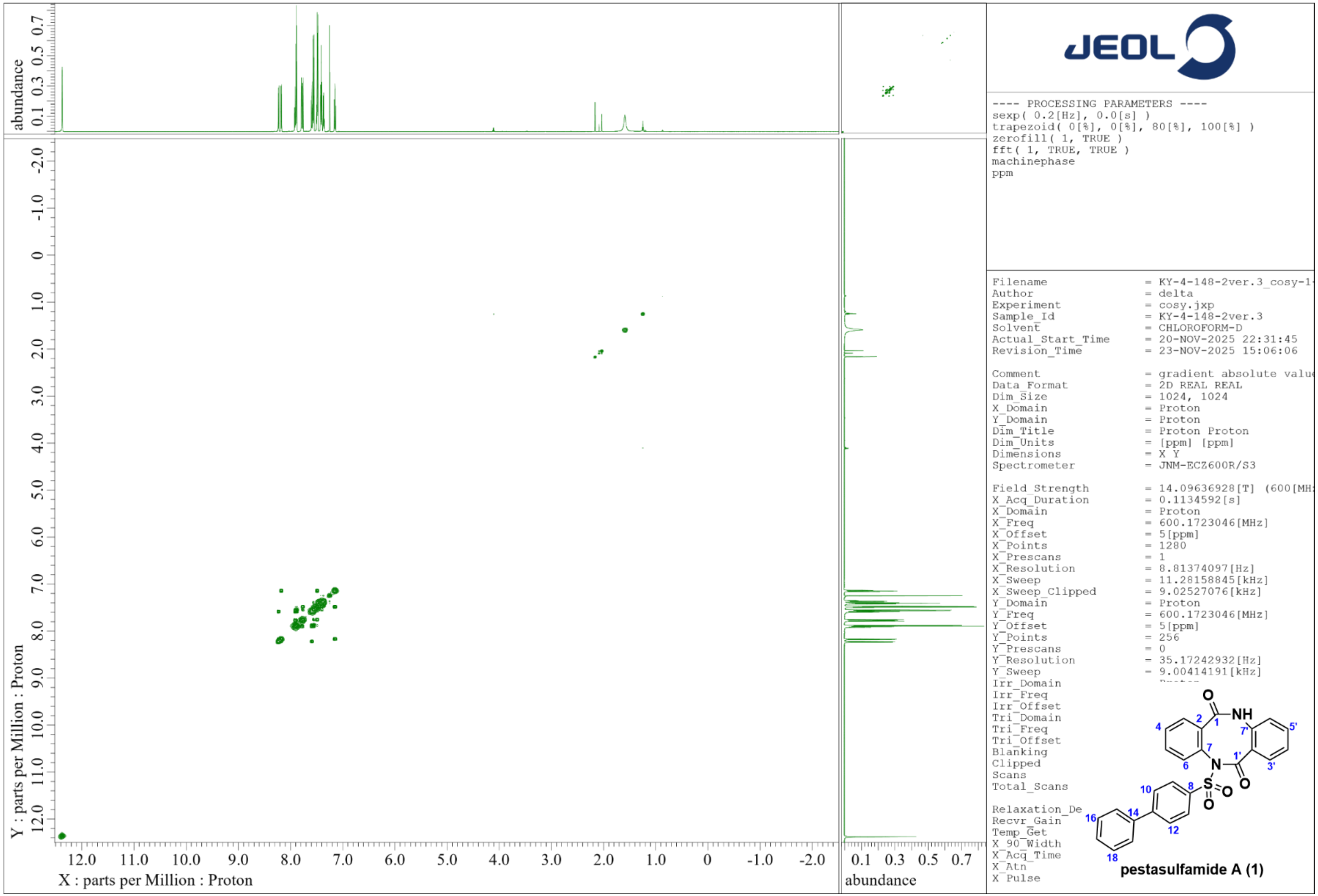
JEOL
abundance
0.1 0.3 0.5 0.7
Y : parts per Million : Proton
-2.0 -1.0 0 1.0 2.0 3.0 4.0 5.0 6.0 7.0 8.0 9.0 10.0 11.0 12.0
X : parts per Million : Proton
12.0 11.0 10.0 9.0 8.0 7.0 6.0 5.0 4.0 3.0 2.0 1.0 0 -1.0 -2.0
0.1 0.3 0.5 0.7
abundance
---- PROCESSING PARAMETERS ----
sexp(0.2[Hz], 0.0[s])
trapezoid(0[%], 0[%], 80[%], 100[%])
zerofill(1, TRUE)
fft(1, TRUE, TRUE)
machinephase
ppm
Filename = KY-4-148-2ver.3_cosy-1
Author = delta
Experiment = cosy.jxp
Sample_Id = KY-4-148-2ver.3
Solvent = CHLOROFORM-D
Actual_Start_Time = 20-NOV-2025 22:31:45
Revision_Time = 23-NOV-2025 15:06:06
Comment = gradient absolute valu
Data_Format = 2D REAL REAL
Dim_Size = 1024, 1024
X_Domain = Proton
Y_Domain = Proton
Dim_Title = Proton Proton
Dim_Units = [ppm] [ppm]
Dimensions = X Y
Spectrometer = JNM-ECZ600R/S3
Field_Strength = 14.09636928[T] (600[MH
X_Acq_Duration = 0.1134592[s]
X_Domain = Proton
X_Freq = 600.1723046[MHz]
X_Offset = 5[ppm]
X_Points = 1280
X_Prescans = 1
X_Resolution = 8.81374097[Hz]
X_Sweep = 11.28158845[kHz]
X_Sweep_Clipped = 9.02527076[kHz]
Y_Domain = Proton
Y_Freq = 600.1723046[MHz]
Y_Offset = 5[ppm]
Y_Points = 256
Y_Prescans = 0
Y_Resolution = 35.17242932[Hz]
Y_Sweep = 9.00414191[kHz]
Irr_Domain
Irr_Freq
Irr_Offset
Tri_Domain
Tri_Freq
Tri_Offset
Blanking
Clipped
Scans
Total_Scans
Relaxation_De
Recvr_Gain
Temp_Get
X_90_Width
X_Acq_Time
X_Atn
X_Pulse
pestasulfamide A (1)

z

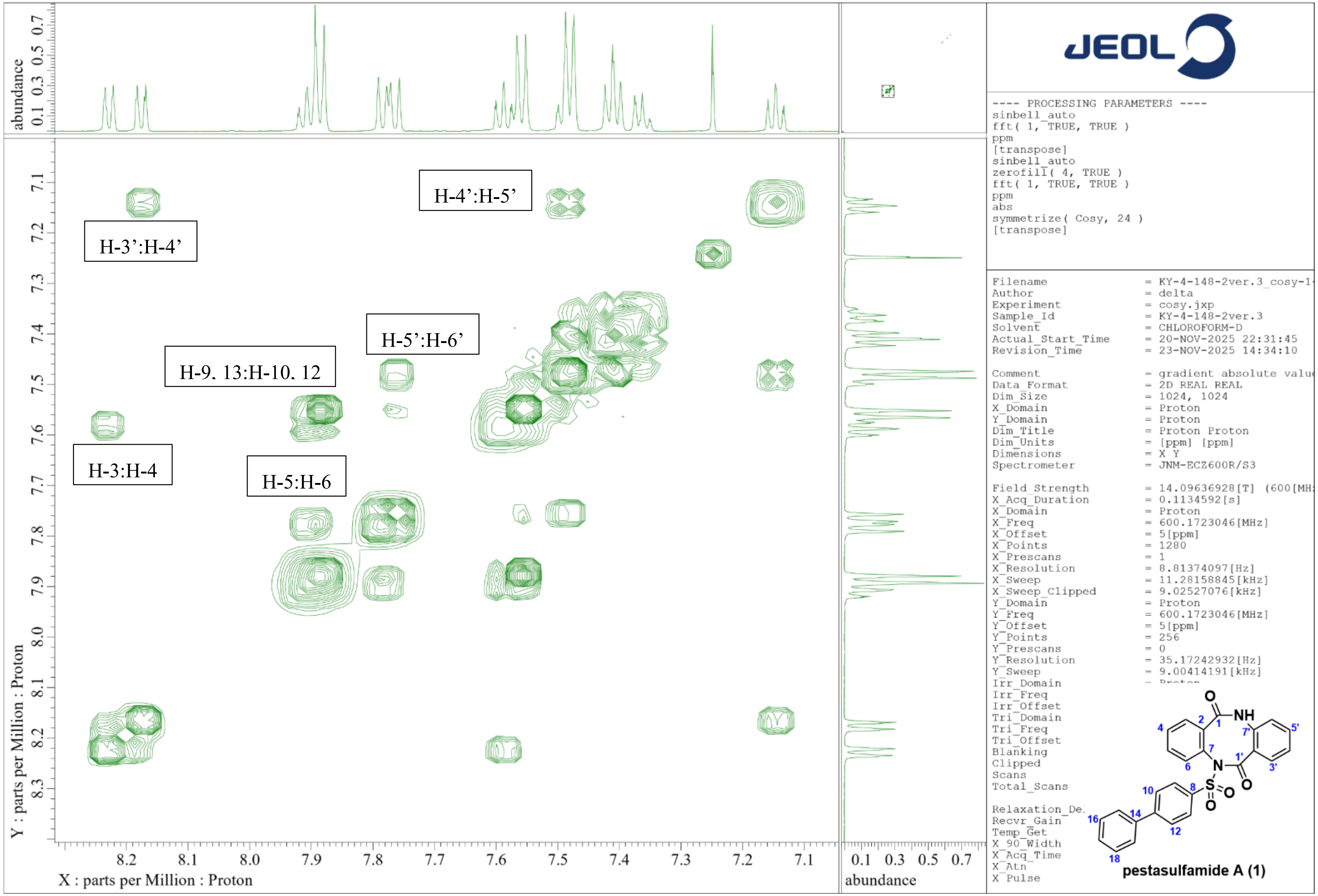
H-4’:H-5’
H-3’:H-4’
H-5’:H-6’
H-9, 13:H-10, 12
H-3:H-4
H-5:H-6
abundance
0.1 0.3 0.5 0.7
Y : parts per Million : Proton
X : parts per Million : Proton
JEOL
---- PROCESSING PARAMETERS ----
sinbell_auto
fft(1, TRUE, TRUE)
ppm
[transpose]
sinbell_auto
zerofill(4, TRUE)
fft(1, TRUE, TRUE)
ppm
abs
symmetrize(Cosy, 24)
[transpose]
Filename = KY-4-148-2ver.3_cosy-1
Author = delta
Experiment = cosy.jxp
Sample_Id = KY-4-148-2ver.3
Solvent = CHLOROFORM-D
Actual_Start_Time = 20-NOV-2025 22:31:45
Revision_Time = 23-NOV-2025 14:34:10
Comment = gradient absolute valu
Data_Format = 2D REAL REAL
Dim_Size = 1024, 1024
X_Domain = Proton
Y_Domain = Proton
Dim_Title = Proton Proton
Dim_Units = [ppm] [ppm]
Dimensions = X Y
Spectrometer = JNM-ECZ600R/S3
Field_Strength = 14.09636928[T] (600[MH
X_Acq_Duration = 0.1134592[s]
X_Domain = Proton
X_Freq = 600.1723046[MHz]
X_Offset = 5[ppm]
X_Points = 1280
X_Prescans = 1
X_Resolution = 8.81374097[Hz]
X_Sweep = 11.28158845[kHz]
X_Sweep_Clipped = 9.02527076[kHz]
Y_Domain = Proton
Y_Freq = 600.1723046[MHz]
Y_Offset = 5[ppm]
Y_Points = 256
Y_Prescans = 0
Y_Resolution = 35.17242932[Hz]
Y_Sweep = 9.00414191[kHz]
Irr_Domain
Irr_Freq
Irr_Offset
Tri_Domain
Tri_Freq
Tri_Offset
Blanking
Clipped
Scans
Total_Scans
Relaxation_De
Recvr_Gain
Temp_Get
X_90_Width
X_Acq_Time
X_Atn
X_Pulse
pestasulfamide A (1)

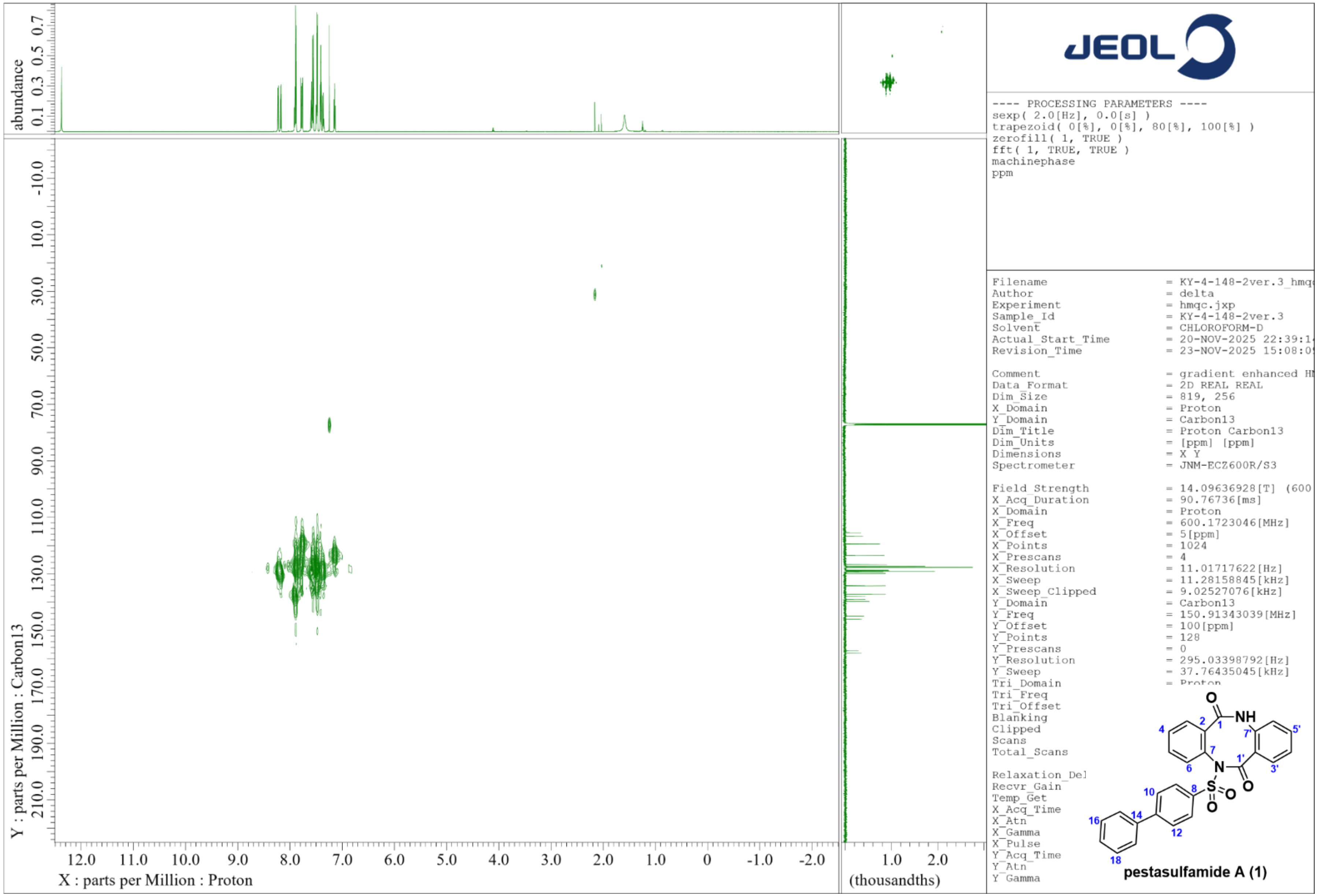
abundance
0.1 0.3 0.5 0.7
Y : parts per Million : Carbon13
-10.0 10.0 30.0 50.0 70.0 90.0 110.0 130.0 150.0 170.0 190.0 210.0
12.0 11.0 10.0 9.0 8.0 7.0 6.0 5.0 4.0 3.0 2.0 1.0 0 -1.0 -2.0
X : parts per Million : Proton
1.0 2.0
(thousandths)
JEOL
---- PROCESSING PARAMETERS ----
sexp(2.0[Hz], 0.0[s])
trapezoid(0[%], 0[%], 80[%], 100[%])
zerofill(1, TRUE)
fft(1, TRUE, TRUE)
machinephase
ppm
Filename = KY-4-148-2ver.3_hmq
Author = delta
Experiment = hmqc.jxp
Sample_Id = KY-4-148-2ver.3
Solvent = CHLOROFORM-D
Actual_Start_Time = 20-NOV-2025 22:39:1
Revision_Time = 23-NOV-2025 15:08:0
Comment = gradient enhanced HM
Data_Format = 2D REAL REAL
Dim_Size = 819, 256
X_Domain = Proton
Y_Domain = Carbon13
Dim_Title = Proton Carbon13
Dim_Units = [ppm] [ppm]
Dimensions = X Y
Spectrometer = JNM-ECZ600R/S3
Field_Strength = 14.09636928[T] (600
X_Acq_Duration = 90.76736[ms]
X_Domain = Proton
X_Freq = 600.1723046[MHz]
X_Offset = 5[ppm]
X_Points = 1024
X_Prescans = 4
X_Resolution = 11.01717622[Hz]
X_Sweep = 11.28158845[kHz]
X_Sweep_Clipped = 9.02527076[kHz]
Y_Domain = Carbon13
Y_Freq = 150.91343039[MHz]
Y_Offset = 100[ppm]
Y_Points = 128
Y_Prescans = 0
Y_Resolution = 295.03398792[Hz]
Y_Sweep = 37.76435045[kHz]
Tri_Domain = Proton
Tri_Freq
Tri_Offset
Blanking
Clipped
Scans
Total_Scans
Relaxation_Del
Recvr_Gain
Temp_Get
X_Acq_Time
X_Atn
X_Gamma
X_Pulse
Y_Acq_Time
Y_Atn
Y_Gamma
pestasulfamide A (1)

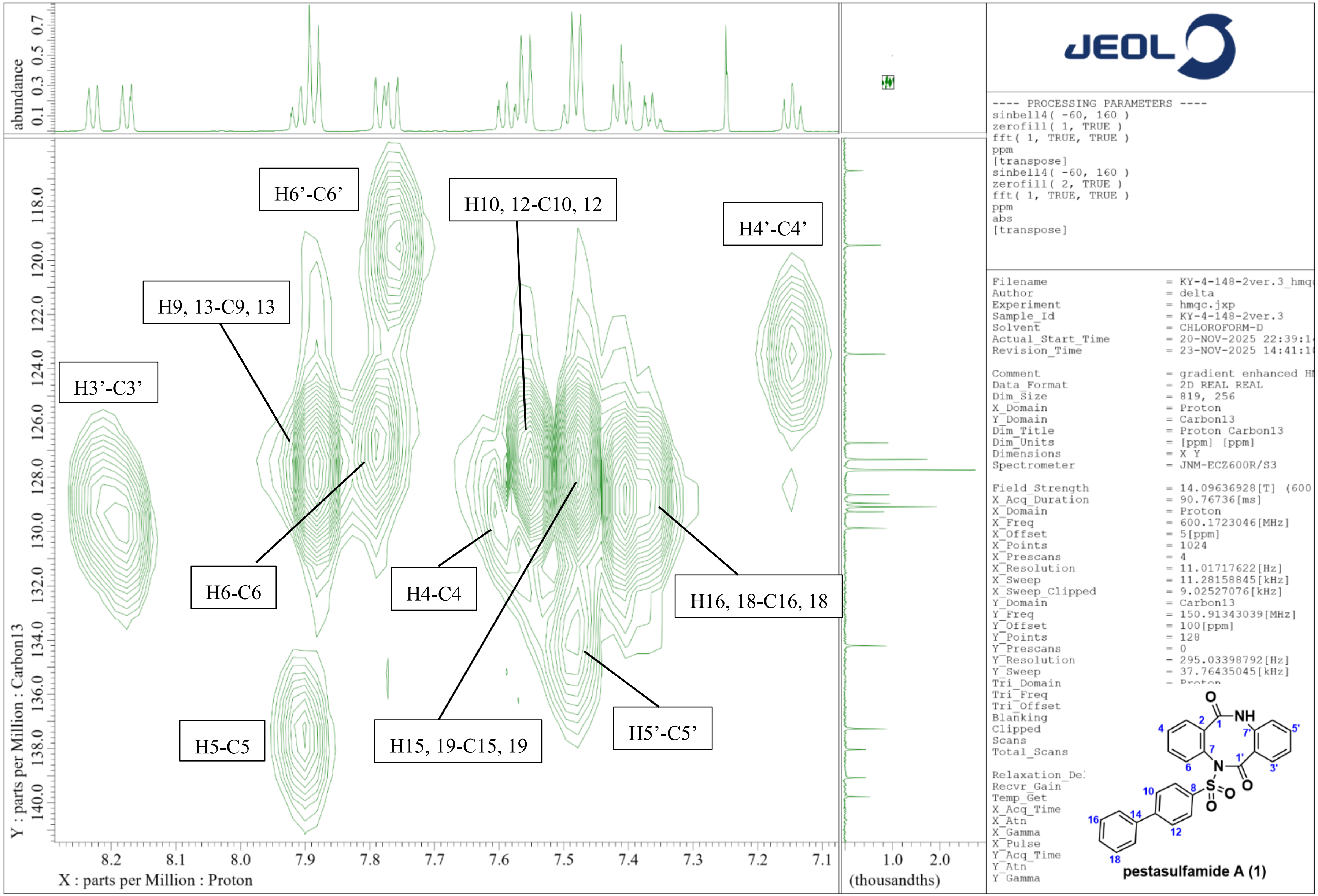
abundance
H6'-C6'
H10, 12-C10, 12
H4'-C4'
H9, 13-C9, 13
H3'-C3'
H6-C6
H4-C4
H16, 18-C16, 18
H5-C5
H15, 19-C15, 19
H5'-C5'
Y : parts per Million : Carbon13
X : parts per Million : Proton
(thousandths)
JEOL
---- PROCESSING PARAMETERS ----
sinbell4(-60, 160)
zerofill(1, TRUE)
fft(1, TRUE, TRUE)
ppm
[transpose]
sinbell4(-60, 160)
zerofill(2, TRUE)
fft(1, TRUE, TRUE)
ppm
abs
[transpose]
Filename = KY-4-148-2ver.3_hmq
Author = delta
Experiment = hmqc.jxp
Sample_Id = KY-4-148-2ver.3
Solvent = CHLOROFORM-D
Actual_Start_Time = 20-NOV-2025 22:39:1
Revision_Time = 23-NOV-2025 14:41:1
Comment = gradient enhanced H
Data_Format = 2D REAL REAL
Dim_Size = 819, 256
X_Domain = Proton
Y_Domain = Carbon13
Dim_Title = Proton Carbon13
Dim_Units = [ppm] [ppm]
Dimensions = X Y
Spectrometer = JNM-ECZ600R/S3
Field_Strength = 14.09636928[T] (600
X_Acq_Duration = 90.76736[ms]
X_Domain = Proton
X_Freq = 600.1723046[MHz]
X_Offset = 5[ppm]
X_Points = 1024
X_Prescans = 4
X_Resolution = 11.01717622[Hz]
X_Sweep = 11.28158845[kHz]
X_Sweep_Clipped = 9.02527076[kHz]
Y_Domain = Carbon13
Y_Freq = 150.91343039[MHz]
Y_Offset = 100[ppm]
Y_Points = 128
Y_Prescans = 0
Y_Resolution = 295.03398792[Hz]
Y_Sweep = 37.76435045[kHz]
Tri_Domain
Tri_Freq
Tri_Offset
Blanking
Clipped
Scans
Total_Scans
Relaxation_De
Recvr_Gain
Temp_Get
X_Acq_Time
X_Atn
X_Gamma
X_Pulse
Y_Acq_Time
Y_Atn
Y_Gamma
pestasulfamide A (1)

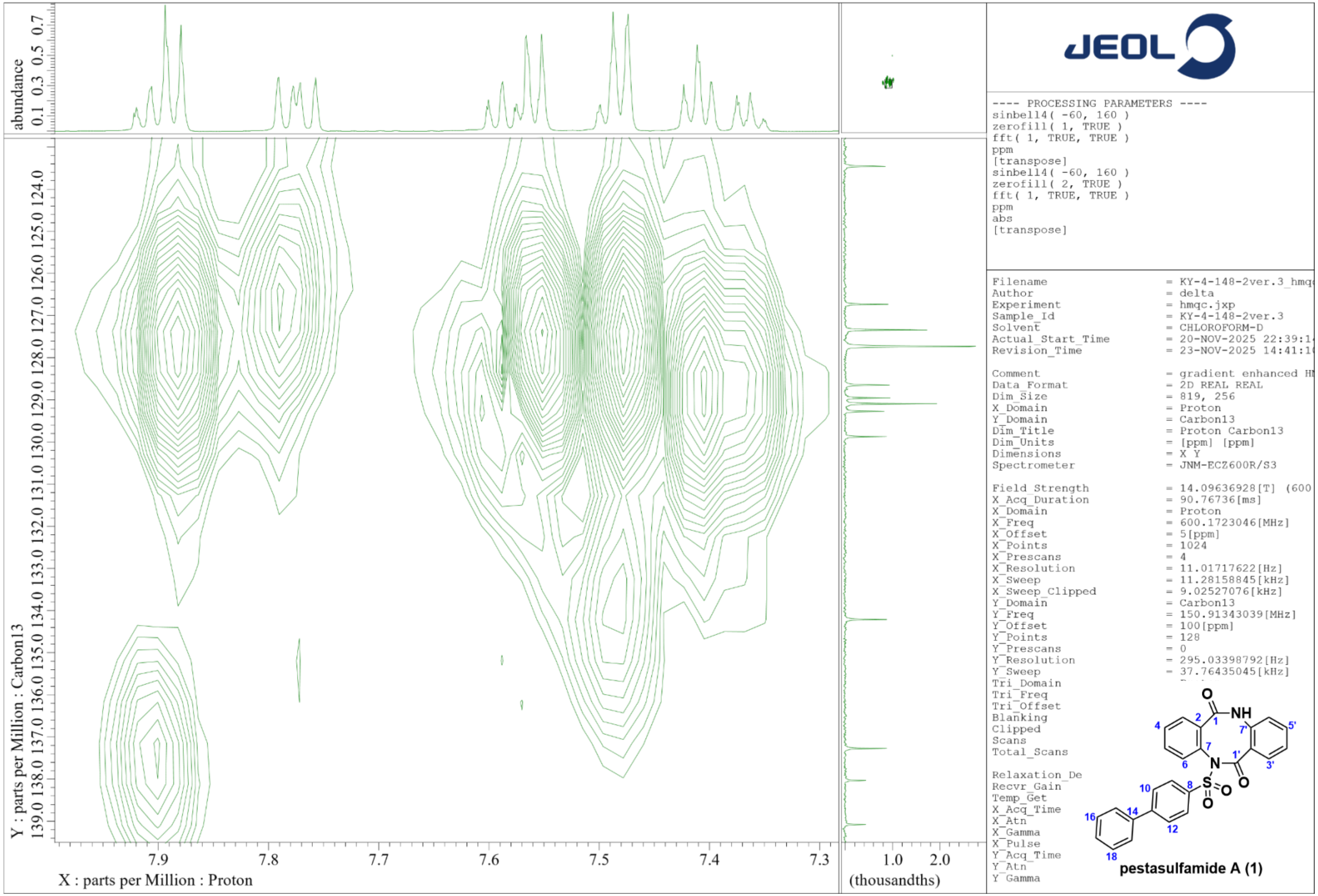
abundance
0.1 0.3 0.5 0.7
Y : parts per Million : Carbon13
124.0 125.0 126.0 127.0 128.0 129.0 130.0 131.0 132.0 133.0 134.0 135.0 136.0 137.0 138.0 139.0
7.9 7.8 7.7 7.6 7.5 7.4 7.3
X : parts per Million : Proton
1.0 2.0
(thousandths)
JEOL
---- PROCESSING PARAMETERS ----
sinbell4(-60, 160)
zerofill(1, TRUE)
fft(1, TRUE, TRUE)
ppm
[transpose]
sinbell4(-60, 160)
zerofill(2, TRUE)
fft(1, TRUE, TRUE)
ppm
abs
[transpose]
Filename = KY-4-148-2ver.3_hmq
Author = delta
Experiment = hmqc.jxp
Sample_Id = KY-4-148-2ver.3
Solvent = CHLOROFORM-D
Actual_Start_Time = 20-NOV-2025 22:39:1
Revision_Time = 23-NOV-2025 14:41:1
Comment = gradient enhanced H
Data_Format = 2D REAL REAL
Dim_Size = 819, 256
X_Domain = Proton
Y_Domain = Carbon13
Dim_Title = Proton Carbon13
Dim_Units = [ppm] [ppm]
Dimensions = X Y
Spectrometer = JNM-ECZ600R/S3
Field_Strength = 14.09636928[T] (600
X_Acq_Duration = 90.76736[ms]
X_Domain = Proton
X_Freq = 600.1723046[MHz]
X_Offset = 5[ppm]
X_Points = 1024
X_Prescans = 4
X_Resolution = 11.01717622[Hz]
X_Sweep = 11.28158845[kHz]
X_Sweep_Clipped = 9.02527076[kHz]
Y_Domain = Carbon13
Y_Freq = 150.91343039[MHz]
Y_Offset = 100[ppm]
Y_Points = 128
Y_Prescans = 0
Y_Resolution = 295.03398792[Hz]
Y_Sweep = 37.76435045[kHz]
Tri_Domain
Tri_Freq
Tri_Offset
Blanking
Clipped
Scans
Total_Scans
Relaxation_De
Recvr_Gain
Temp_Get
X_Acq_Time
X_Atn
X_Gamma
X_Pulse
Y_Acq_Time
Y_Atn
Y_Gamma
pestasulfamide A (1)

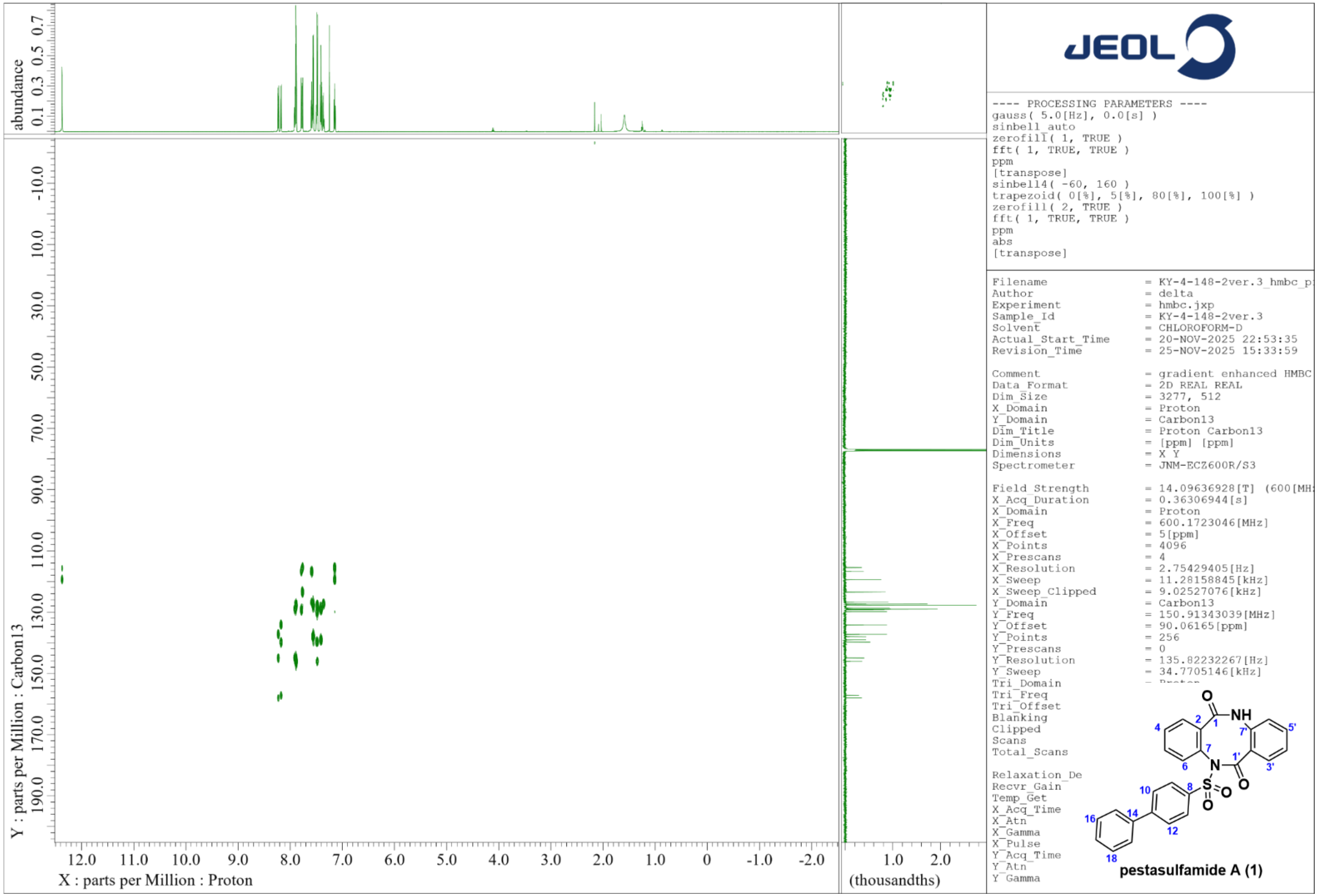

abundance
0.1 0.3 0.5 0.7
Y : parts per Million : Carbon13
-10.0 10.0 30.0 50.0 70.0 90.0 110.0 130.0 150.0 170.0 190.0
X : parts per Million : Proton
12.0 11.0 10.0 9.0 8.0 7.0 6.0 5.0 4.0 3.0 2.0 1.0 0 -1.0 -2.0
1.0 2.0
(thousandths)
---- PROCESSING PARAMETERS ----
gauss(5.0[Hz], 0.0[s])
sinbell_auto
zerofill(1, TRUE)
fft(1, TRUE, TRUE)
ppm
[transpose]
sinbell4(-60, 160)
trapezoid(0[%], 5[%], 80[%], 100[%])
zerofill(2, TRUE)
fft(1, TRUE, TRUE)
ppm
abs
[transpose]
Filename = KY-4-148-2ver.3_hmbc_p
Author = delta
Experiment = hmbc.jxp
Sample_Id = KY-4-148-2ver.3
Solvent = CHLOROFORM-D
Actual_Start_Time = 20-NOV-2025 22:53:35
Revision_Time = 25-NOV-2025 15:33:59
Comment = gradient enhanced HMBC
Data_Format = 2D REAL REAL
Dim_Size = 3277, 512
X_Domain = Proton
Y_Domain = Carbon13
Dim_Title = Proton Carbon13
Dim_Units = [ppm] [ppm]
Dimensions = X Y
Spectrometer = JNM-ECZ600R/S3
Field_Strength = 14.09636928[T] (600[MH
X_Acq_Duration = 0.36306944[s]
X_Domain = Proton
X_Freq = 600.1723046[MHz]
X_Offset = 5[ppm]
X_Points = 4096
X_Prescans = 4
X_Resolution = 2.75429405[Hz]
X_Sweep = 11.28158845[kHz]
X_Sweep_Clipped = 9.02527076[kHz]
Y_Domain = Carbon13
Y_Freq = 150.91343039[MHz]
Y_Offset = 90.06165[ppm]
Y_Points = 256
Y_Prescans = 0
Y_Resolution = 135.82232267[Hz]
Y_Sweep = 34.7705146[kHz]
Tri_Domain
Tri_Freq
Tri_Offset
Blanking
Clipped
Scans
Total_Scans
Relaxation_De
Recvr_Gain
Temp_Get
X_Acq_Time
X_Atn
X_Gamma
X_Pulse
Y_Acq_Time
Y_Atn
Y_Gamma
O
NH
O
S
O
O
N
1 2 4 6 7 7' 1' 3' 5' 8 10 12 14 16 18
pestasulfamide A (1)

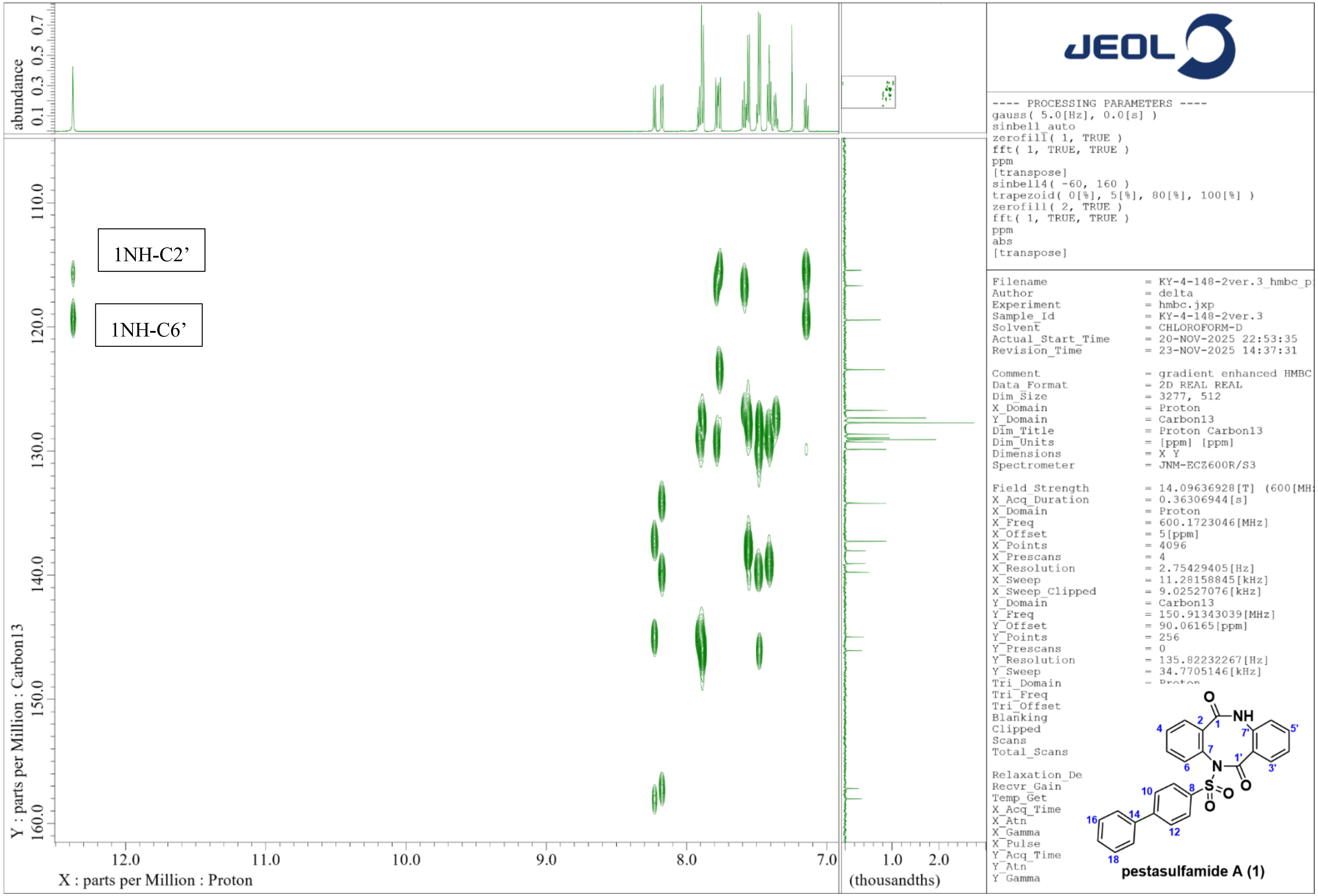

JEOL
1NH-C2’
1NH-C6’
abundance
X : parts per Million : Proton
Y : parts per Million : Carbon13
(thousandths)
---- PROCESSING PARAMETERS ----
gauss(5.0[Hz], 0.0[s])
sinbell_auto
zerofill(1, TRUE)
fft(1, TRUE, TRUE)
ppm
[transpose]
sinbell4(-60, 160)
trapezoid(0[%], 5[%], 80[%], 100[%])
zerofill(2, TRUE)
fft(1, TRUE, TRUE)
ppm
abs
[transpose]
Filename = KY-4-148-2ver.3_hmbc_p
Author = delta
Experiment = hmbc.jxp
Sample_Id = KY-4-148-2ver.3
Solvent = CHLOROFORM-D
Actual_Start_Time = 20-NOV-2025 22:53:35
Revision_Time = 23-NOV-2025 14:37:31
Comment = gradient enhanced HMBC
Data_Format = 2D REAL REAL
Dim_Size = 3277, 512
X_Domain = Proton
Y_Domain = Carbon13
Dim_Title = Proton Carbon13
Dim_Units = [ppm] [ppm]
Dimensions = X Y
Spectrometer = JNM-ECZ600R/S3
Field_Strength = 14.09636928[T] (600[MH
X_Acq_Duration = 0.36306944[s]
X_Domain = Proton
X_Freq = 600.1723046[MHz]
X_Offset = 5[ppm]
X_Points = 4096
X_Prescans = 4
X_Resolution = 2.75429405[Hz]
X_Sweep = 11.28158845[kHz]
X_Sweep_Clipped = 9.02527076[kHz]
Y_Domain = Carbon13
Y_Freq = 150.91343039[MHz]
Y_Offset = 90.06165[ppm]
Y_Points = 256
Y_Prescans = 0
Y_Resolution = 135.82232267[Hz]
Y_Sweep = 34.7705146[kHz]
Tri_Domain
Tri_Freq
Tri_Offset
Blanking
Clipped
Scans
Total_Scans
Relaxation_De
Recvr_Gain
Temp_Get
X_Acq_Time
X_Atn
X_Gamma
X_Pulse
Y_Acq_Time
Y_Atn
Y_Gamma
pestasulfamide A (1)

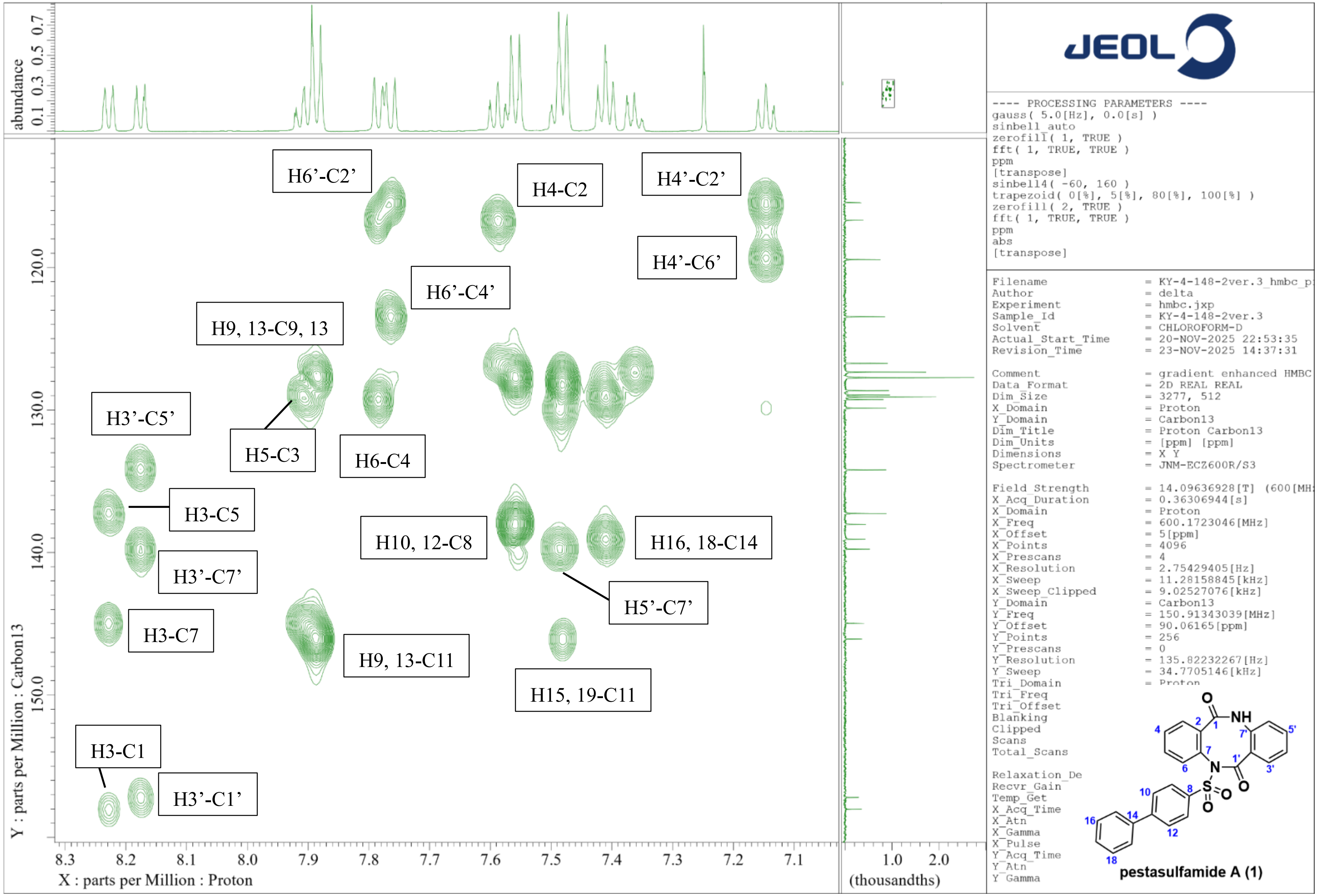
abundance
0.1 0.3 0.5 0.7
H6'-C2'
H4-C2
H4'-C2'
H4'-C6'
H6'-C4'
H9, 13-C9, 13
H3'-C5'
H5-C3
H6-C4
H3-C5
H10, 12-C8
H16, 18-C14
H3'-C7'
H5'-C7'
H3-C7
H9, 13-C11
H15, 19-C11
H3-C1
H3'-C1'
Y : parts per Million : Carbon13
120.0
130.0
140.0
150.0
8.3 8.2 8.1 8.0 7.9 7.8 7.7 7.6 7.5 7.4 7.3 7.2 7.1
X : parts per Million : Proton
1.0 2.0
(thousandths)
JEOL
---- PROCESSING PARAMETERS ----
gauss(5.0[Hz], 0.0[s])
sinbell_auto
zerofill(1, TRUE)
fft(1, TRUE, TRUE)
ppm
[transpose]
sinbell4(-60, 160)
trapezoid(0[%], 5[%], 80[%], 100[%])
zerofill(2, TRUE)
fft(1, TRUE, TRUE)
ppm
abs
[transpose]
Filename = KY-4-148-2ver.3_hmbc_p
Author = delta
Experiment = hmbc.jxp
Sample_Id = KY-4-148-2ver.3
Solvent = CHLOROFORM-D
Actual_Start_Time = 20-NOV-2025 22:53:35
Revision_Time = 23-NOV-2025 14:37:31
Comment = gradient enhanced HMBC
Data_Format = 2D REAL REAL
Dim_Size = 3277, 512
X_Domain = Proton
Y_Domain = Carbon13
Dim_Title = Proton Carbon13
Dim_Units = [ppm] [ppm]
Dimensions = X Y
Spectrometer = JNM-ECZ600R/S3
Field_Strength = 14.09636928[T] (600[MH
X_Acq_Duration = 0.36306944[s]
X_Domain = Proton
X_Freq = 600.1723046[MHz]
X_Offset = 5[ppm]
X_Points = 4096
X_Prescans = 4
X_Resolution = 2.75429405[Hz]
X_Sweep = 11.28158845[kHz]
X_Sweep_Clipped = 9.02527076[kHz]
Y_Domain = Carbon13
Y_Freq = 150.91343039[MHz]
Y_Offset = 90.06165[ppm]
Y_Points = 256
Y_Prescans = 0
Y_Resolution = 135.82232267[Hz]
Y_Sweep = 34.7705146[kHz]
Tri_Domain = Proton
Tri_Freq
Tri_Offset
Blanking
Clipped
Scans
Total_Scans
Relaxation_De
Recvr_Gain
Temp_Get
X_Acq_Time
X_Atn
X_Gamma
X_Pulse
Y_Acq_Time
Y_Atn
Y_Gamma
pestasulfamide A (1)

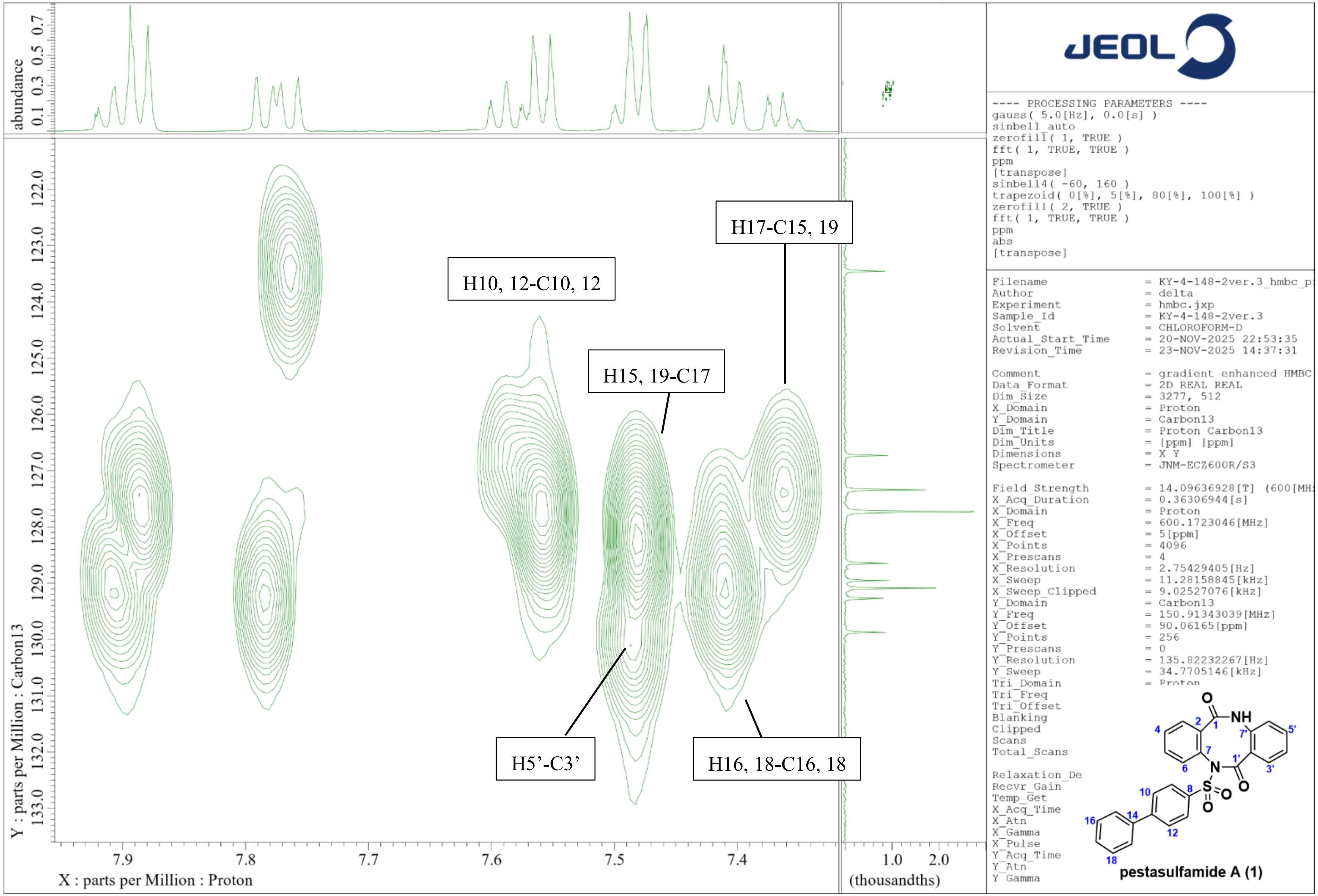

abundance
0.1
0.3
0.5
0.7
JEOL
---- PROCESSING PARAMETERS ----
gauss(5.0[Hz], 0.0[s])
sinbell_auto
zerofill(1, TRUE)
fft(1, TRUE, TRUE)
ppm
[transpose]
sinbell4(-60, 160)
trapezoid(0[%], 5[%], 80[%], 100[%])
zerofill(2, TRUE)
fft(1, TRUE, TRUE)
ppm
abs
[transpose]
Filename = KY-4-148-2ver.3_hmbc_p
Author = delta
Experiment = hmbc.jxp
Sample_Id = KY-4-148-2ver.3
Solvent = CHLOROFORM-D
Actual_Start_Time = 20-NOV-2025 22:53:35
Revision_Time = 23-NOV-2025 14:37:31
Comment = gradient enhanced HMBC
Data_Format = 2D REAL REAL
Dim_Size = 3277, 512
X_Domain = Proton
Y_Domain = Carbon13
Dim_Title = Proton Carbon13
Dim_Units = [ppm] [ppm]
Dimensions = X Y
Spectrometer = JNM-ECZ600R/S3
Field_Strength = 14.09636928[T] (600[MH
X_Acq_Duration = 0.36306944[s]
X_Domain = Proton
X_Freq = 600.1723046[MHz]
X_Offset = 5[ppm]
X_Points = 4096
X_Prescans = 4
X_Resolution = 2.75429405[Hz]
X_Sweep = 11.28158845[kHz]
X_Sweep_Clipped = 9.02527076[kHz]
Y_Domain = Carbon13
Y_Freq = 150.91343039[MHz]
Y_Offset = 90.06165[ppm]
Y_Points = 256
Y_Prescans = 0
Y_Resolution = 135.82232267[Hz]
Y_Sweep = 34.7705146[kHz]
Tri_Domain = Proton
Tri_Freq
Tri_Offset
Blanking
Clipped
Scans
Total_Scans
Relaxation_De
Recvr_Gain
Temp_Get
X_Acq_Time
X_Atn
X_Gamma
X_Pulse
Y_Acq_Time
Y_Atn
Y_Gamma
H17-C15, 19
H10, 12-C10, 12
H15, 19-C17
H5'-C3'
H16, 18-C16, 18
Y : parts per Million : Carbon13
122.0
123.0
124.0
125.0
126.0
127.0
128.0
129.0
130.0
131.0
132.0
133.0
7.9
7.8
7.7
7.6
7.5
7.4
X : parts per Million : Proton
1.0
2.0
(thousandths)
O
NH
N
S
O
O
O
1
2
4
6
7
1'
3'
5'
7'
8
10
12
14
16
18
pestasulfamide A (1)

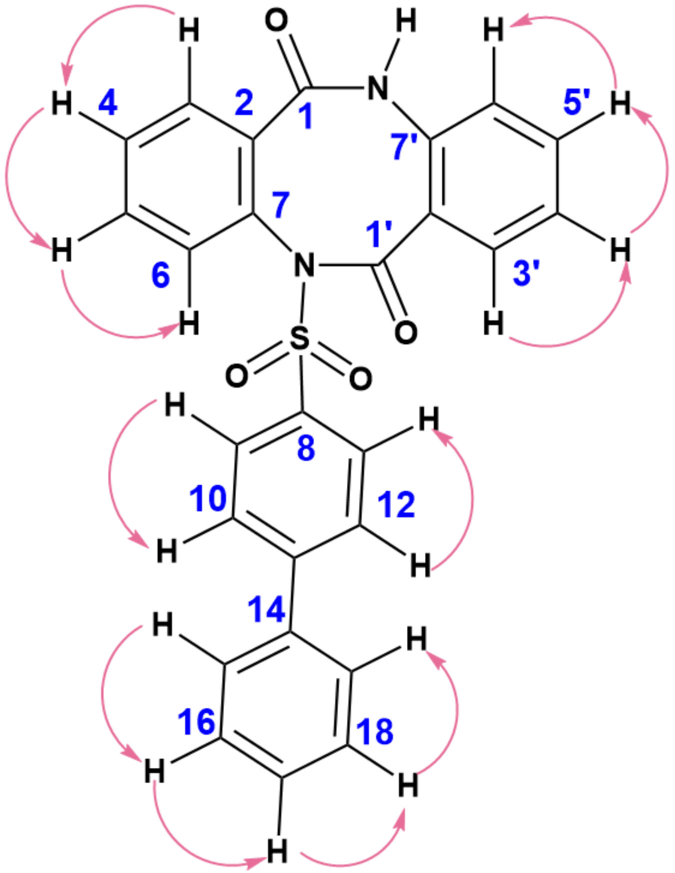

Figure S1. COSY correlations of pestasulfamide A (**1**).

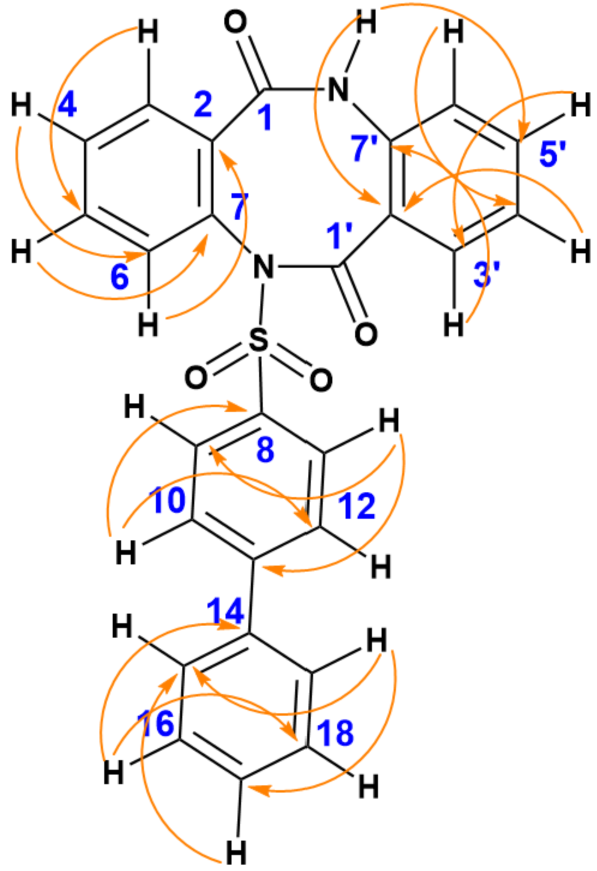

Figure S2. HMBC correlations of pestasulfamide A (**1**).

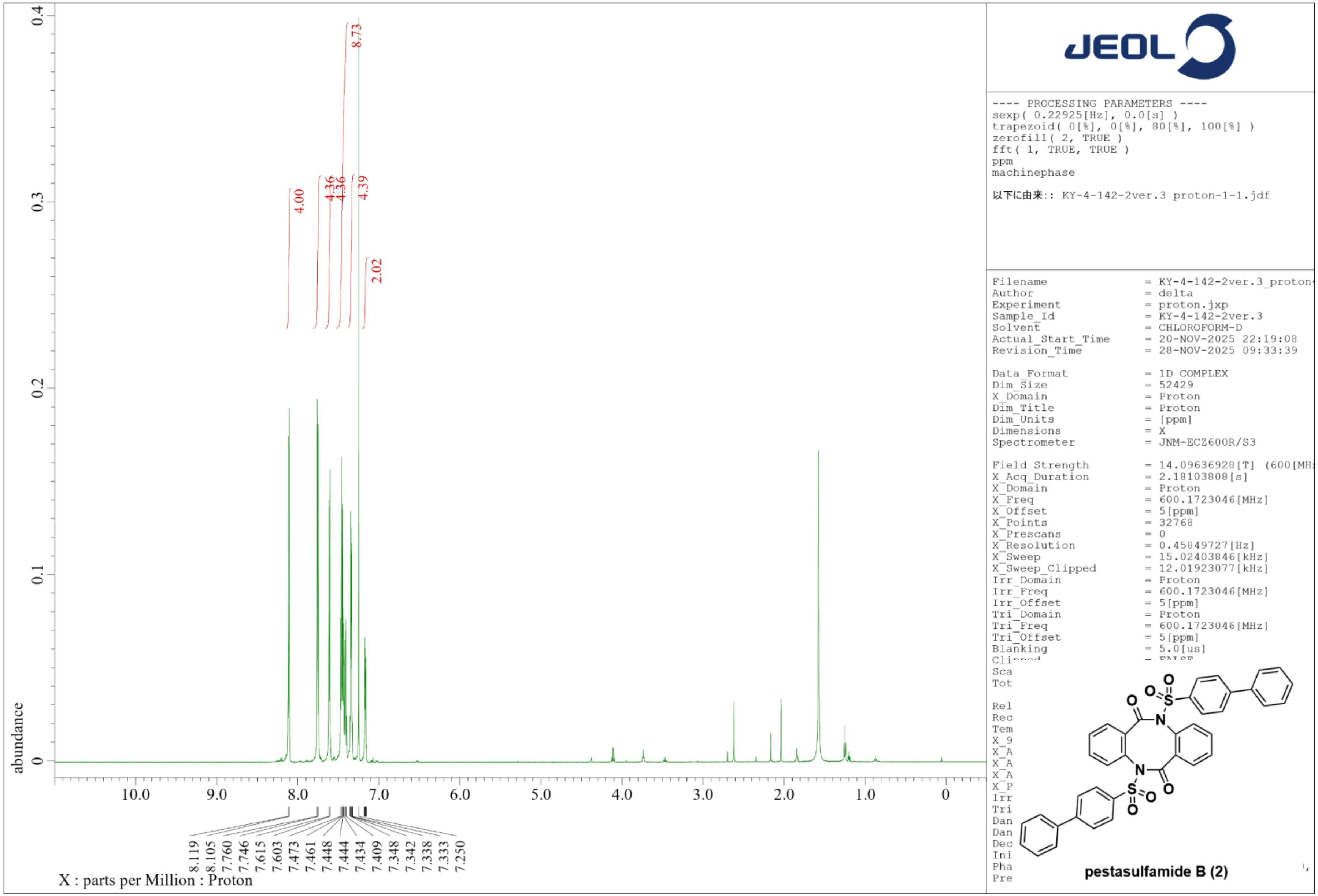
abundance
0.4
0.3
0.2
0.1
0
4.00
4.36
4.36
8.73
4.39
2.02
10.0
9.0
8.0
7.0
6.0
5.0
4.0
3.0
2.0
1.0
0
8.119
8.105
7.760
7.746
7.615
7.603
7.473
7.461
7.448
7.444
7.434
7.409
7.348
7.342
7.338
7.333
7.250
X : parts per Million : Proton
JEOL
---- PROCESSING PARAMETERS ----
sexp(0.22925[Hz], 0.0[s])
trapezoid(0[%], 0[%], 80[%], 100[%])
zerofill(2, TRUE)
fft(1, TRUE, TRUE)
ppm
machinephase
以下に由来:: KY-4-142-2ver.3 proton-1-1.jdf
Filename = KY-4-142-2ver.3_proton
Author = delta
Experiment = proton.jxp
Sample_Id = KY-4-142-2ver.3
Solvent = CHLOROFORM-D
Actual_Start_Time = 20-NOV-2025 22:19:08
Revision_Time = 28-NOV-2025 09:33:39
Data_Format = 1D COMPLEX
Dim_Size = 52429
X_Domain = Proton
Dim_Title = Proton
Dim_Units = [ppm]
Dimensions = X
Spectrometer = JNM-ECZ600R/S3
Field_Strength = 14.09636928[T] (600[MH
X_Acq_Duration = 2.18103808[s]
X_Domain = Proton
X_Freq = 600.1723046[MHz]
X_Offset = 5[ppm]
X_Points = 32768
X_Prescans = 0
X_Resolution = 0.45849727[Hz]
X_Sweep = 15.02403846[kHz]
X_Sweep_Clipped = 12.01923077[kHz]
Irr_Domain = Proton
Irr_Freq = 600.1723046[MHz]
Irr_Offset = 5[ppm]
Tri_Domain = Proton
Tri_Freq = 600.1723046[MHz]
Tri_Offset = 5[ppm]
Blanking = 5.0[us]
pestasulfamide B (2)

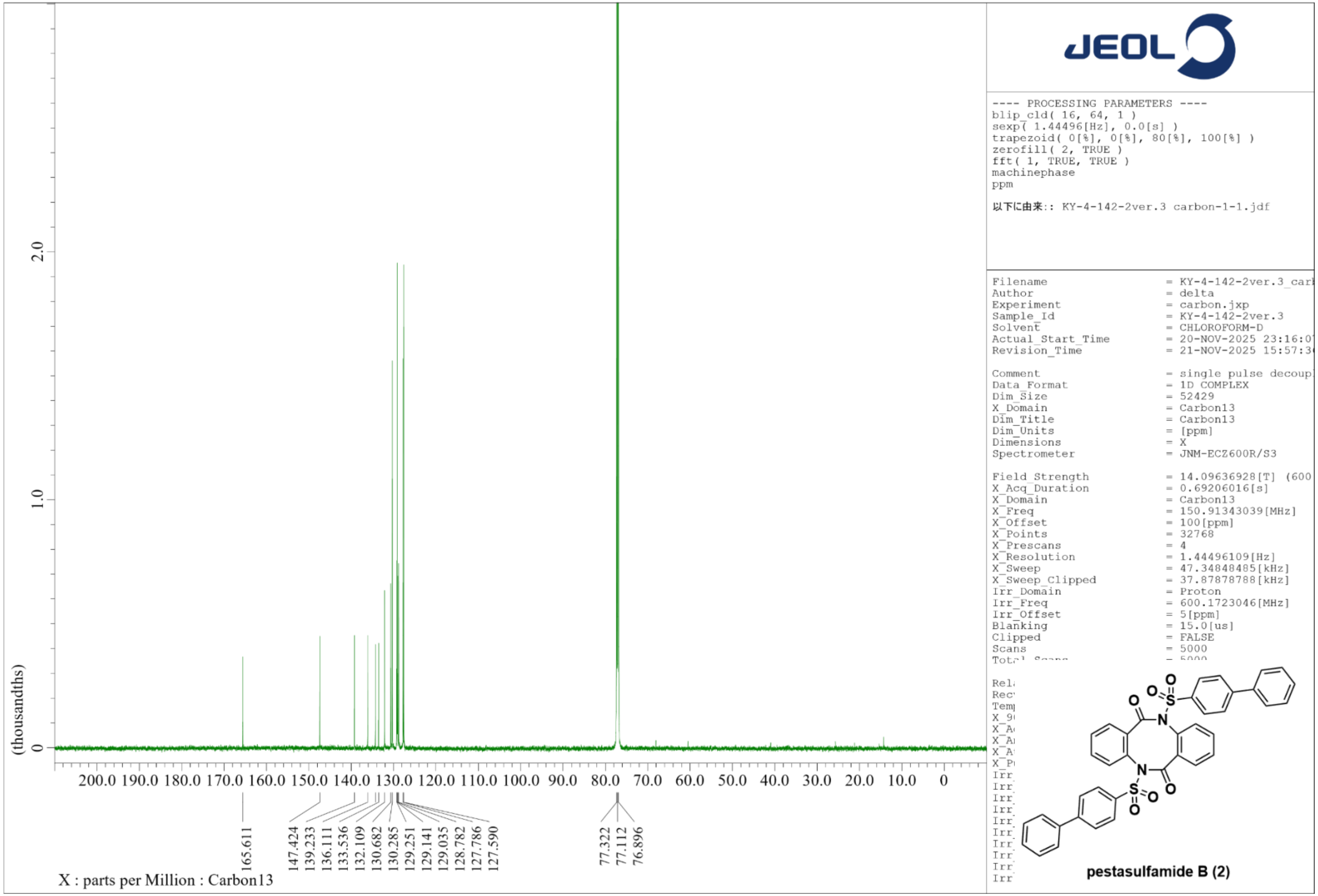
JEOL
---- PROCESSING PARAMETERS ----
blip_cld(16, 64, 1)
sexp(1.44496[Hz], 0.0[s])
trapezoid(0[%], 0[%], 80[%], 100[%])
zerofill(2, TRUE)
fft(1, TRUE, TRUE)
machinephase
ppm
以下に由来:: KY-4-142-2ver.3 carbon-1-1.jdf
Filename = KY-4-142-2ver.3_car
Author = delta
Experiment = carbon.jxp
Sample_Id = KY-4-142-2ver.3
Solvent = CHLOROFORM-D
Actual_Start_Time = 20-NOV-2025 23:16:0
Revision_Time = 21-NOV-2025 15:57:3
Comment = single pulse decoup
Data_Format = 1D COMPLEX
Dim_Size = 52429
X_Domain = Carbon13
Dim_Title = Carbon13
Dim_Units = [ppm]
Dimensions = X
Spectrometer = JNM-ECZ600R/S3
Field_Strength = 14.09636928[T] (600
X_Acq_Duration = 0.69206016[s]
X_Domain = Carbon13
X_Freq = 150.91343039[MHz]
X_Offset = 100[ppm]
X_Points = 32768
X_Prescans = 4
X_Resolution = 1.44496109[Hz]
X_Sweep = 47.34848485[kHz]
X_Sweep_Clipped = 37.87878788[kHz]
Irr_Domain = Proton
Irr_Freq = 600.1723046[MHz]
Irr_Offset = 5[ppm]
Blanking = 15.0[us]
Clipped = FALSE
Scans = 5000
(thousandths)
2.0
1.0
0
200.0 190.0 180.0 170.0 160.0 150.0 140.0 130.0 120.0 110.0 100.0 90.0 80.0 70.0 60.0 50.0 40.0 30.0 20.0 10.0 0
165.611
147.424
139.233
136.111
133.536
132.109
130.682
130.285
129.251
129.141
129.035
128.782
127.786
127.590
77.322
77.112
76.896
X : parts per Million : Carbon13
pestasulfamide B (2)

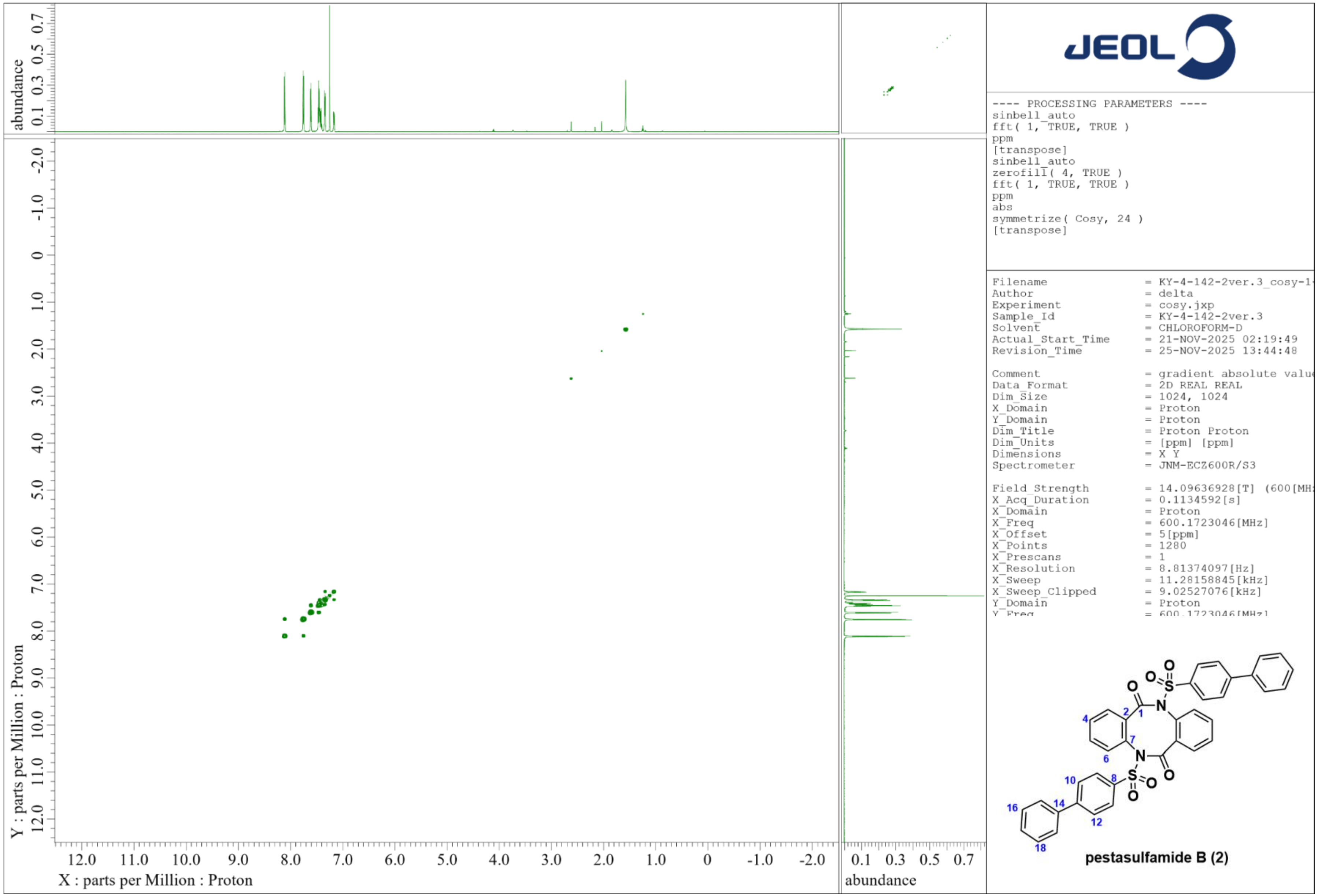
JEOL
---- PROCESSING PARAMETERS ----
sinbell_auto
fft(1, TRUE, TRUE)
ppm
[transpose]
sinbell_auto
zerofill(4, TRUE)
fft(1, TRUE, TRUE)
ppm
abs
symmetrize(Cosy, 24)
[transpose]
Filename = KY-4-142-2ver.3_cosy-1
Author = delta
Experiment = cosy.jxp
Sample_Id = KY-4-142-2ver.3
Solvent = CHLOROFORM-D
Actual_Start_Time = 21-NOV-2025 02:19:49
Revision_Time = 25-NOV-2025 13:44:48
Comment = gradient absolute valu
Data_Format = 2D REAL REAL
Dim_Size = 1024, 1024
X_Domain = Proton
Y_Domain = Proton
Dim_Title = Proton Proton
Dim_Units = [ppm] [ppm]
Dimensions = X Y
Spectrometer = JNM-ECZ600R/S3
Field_Strength = 14.09636928[T] (600[MH
X_Acq_Duration = 0.1134592[s]
X_Domain = Proton
X_Freq = 600.1723046[MHz]
X_Offset = 5[ppm]
X_Points = 1280
X_Prescans = 1
X_Resolution = 8.81374097[Hz]
X_Sweep = 11.28158845[kHz]
X_Sweep_Clipped = 9.02527076[kHz]
Y_Domain = Proton
abundance
X : parts per Million : Proton
Y : parts per Million : Proton
pestasulfamide B (2)

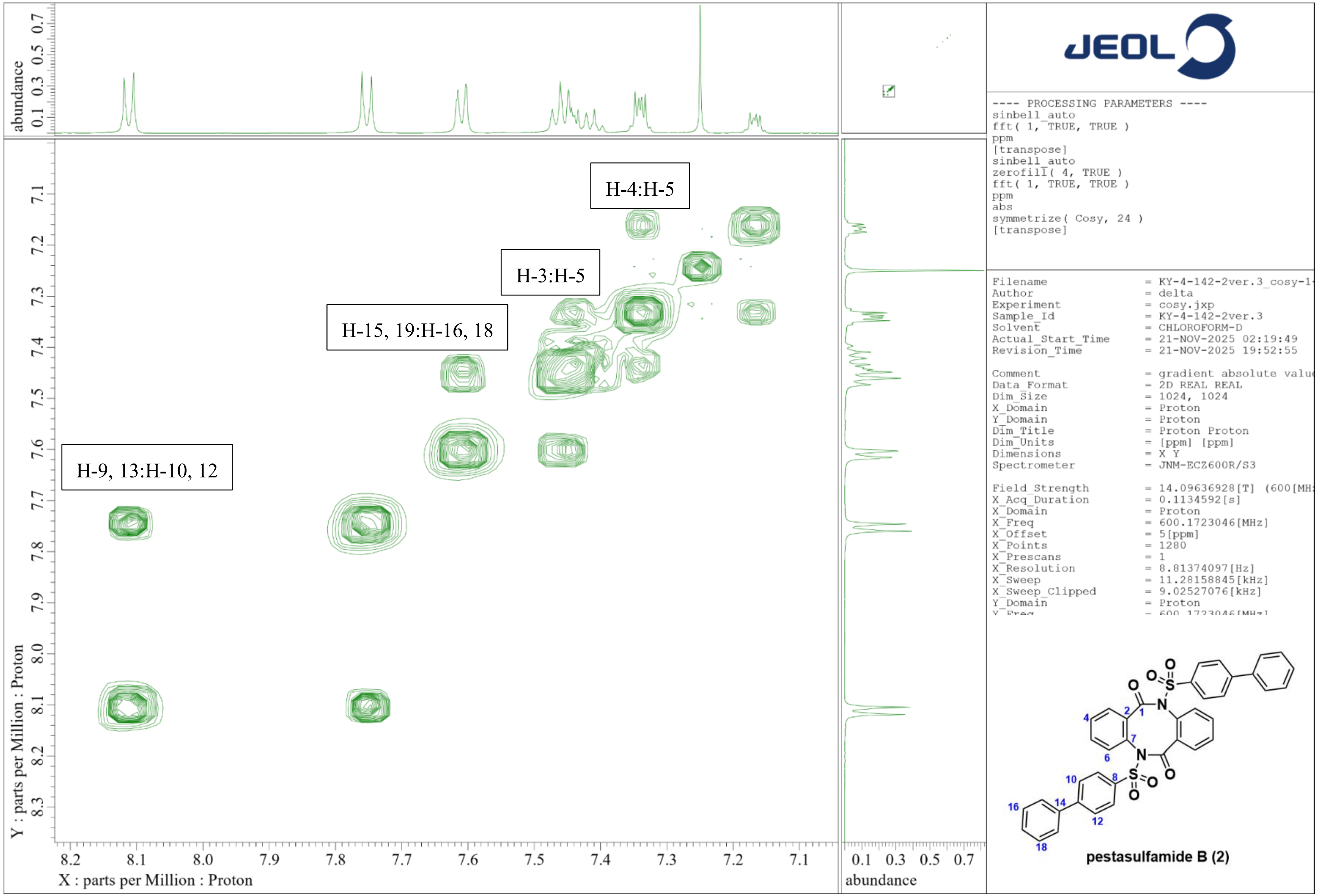
H-4:H-5
H-3:H-5
H-15, 19:H-16, 18
H-9, 13:H-10, 12
abundance
Y : parts per Million : Proton
X : parts per Million : Proton
8.2 8.1 8.0 7.9 7.8 7.7 7.6 7.5 7.4 7.3 7.2 7.1
0.1 0.3 0.5 0.7
JEOL
---- PROCESSING PARAMETERS ----
sinbell_auto
fft(1, TRUE, TRUE)
ppm
[transpose]
sinbell_auto
zerofill(4, TRUE)
fft(1, TRUE, TRUE)
ppm
abs
symmetrize(Cosy, 24)
[transpose]
Filename = KY-4-142-2ver.3_cosy-1
Author = delta
Experiment = cosy.jxp
Sample_Id = KY-4-142-2ver.3
Solvent = CHLOROFORM-D
Actual_Start_Time = 21-NOV-2025 02:19:49
Revision_Time = 21-NOV-2025 19:52:55
Comment = gradient absolute valu
Data_Format = 2D REAL REAL
Dim_Size = 1024, 1024
X_Domain = Proton
Y_Domain = Proton
Dim_Title = Proton Proton
Dim_Units = [ppm] [ppm]
Dimensions = X Y
Spectrometer = JNM-ECZ600R/S3
Field_Strength = 14.09636928[T] (600[MH
X_Acq_Duration = 0.1134592[s]
X_Domain = Proton
X_Freq = 600.1723046[MHz]
X_Offset = 5[ppm]
X_Points = 1280
X_Prescans = 1
X_Resolution = 8.81374097[Hz]
X_Sweep = 11.28158845[kHz]
X_Sweep_Clipped = 9.02527076[kHz]
Y_Domain = Proton
pestasulfamide B (2)

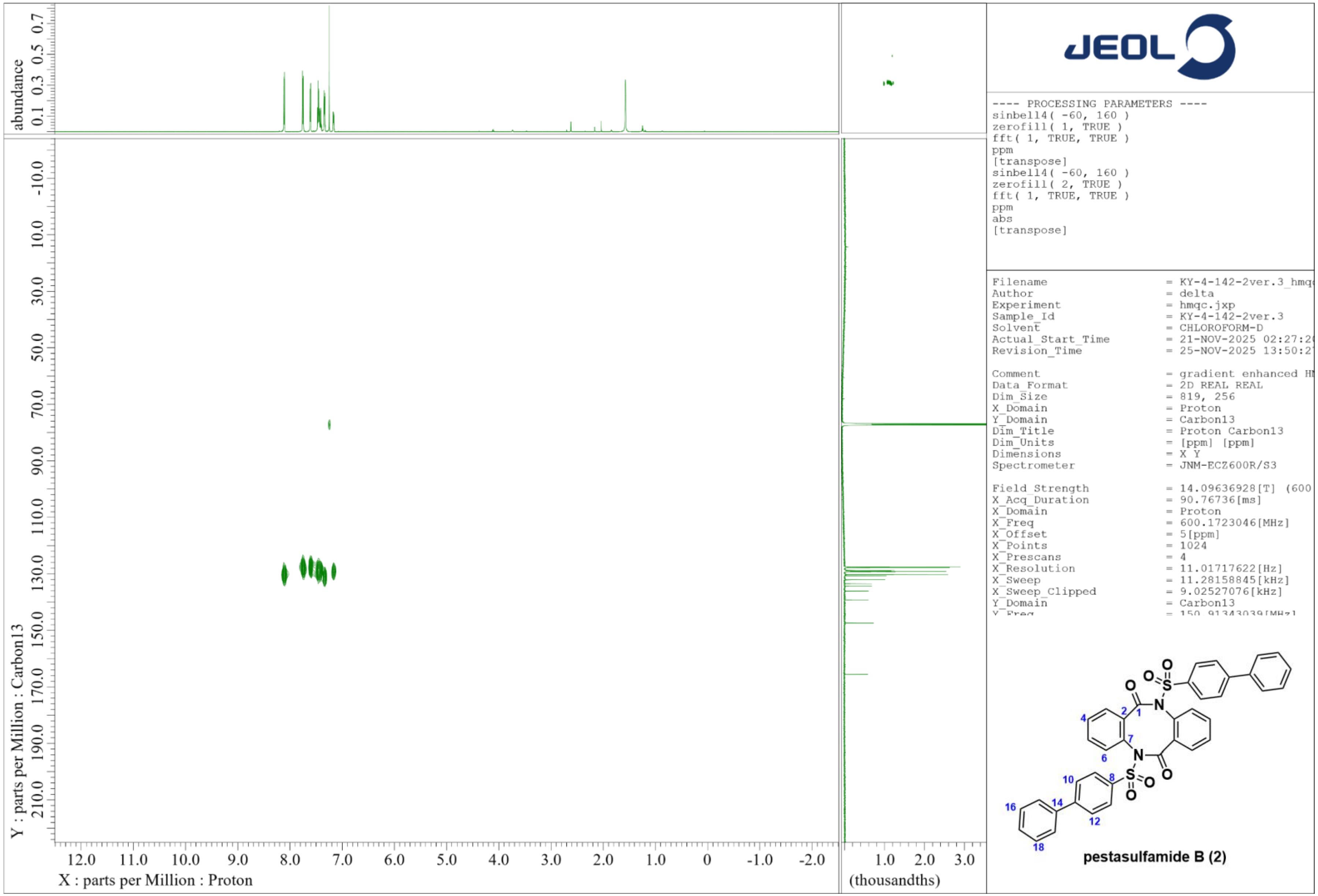
abundance
0.1 0.3 0.5 0.7
---- PROCESSING PARAMETERS ----
sinbell4(-60, 160)
zerofill(1, TRUE)
fft(1, TRUE, TRUE)
ppm
[transpose]
sinbell4(-60, 160)
zerofill(2, TRUE)
fft(1, TRUE, TRUE)
ppm
abs
[transpose]
Filename = KY-4-142-2ver.3_hmq
Author = delta
Experiment = hmqc.jxp
Sample_Id = KY-4-142-2ver.3
Solvent = CHLOROFORM-D
Actual_Start_Time = 21-NOV-2025 02:27:2
Revision_Time = 25-NOV-2025 13:50:2
Comment = gradient enhanced H
Data_Format = 2D REAL REAL
Dim_Size = 819, 256
X_Domain = Proton
Y_Domain = Carbon13
Dim_Title = Proton Carbon13
Dim_Units = [ppm] [ppm]
Dimensions = X Y
Spectrometer = JNM-ECZ600R/S3
Field_Strength = 14.09636928[T] (600
X_Acq_Duration = 90.76736[ms]
X_Domain = Proton
X_Freq = 600.1723046[MHz]
X_Offset = 5[ppm]
X_Points = 1024
X_Prescans = 4
X_Resolution = 11.01717622[Hz]
X_Sweep = 11.28158845[kHz]
X_Sweep_Clipped = 9.02527076[kHz]
Y_Domain = Carbon13
Y : parts per Million : Carbon13
-10.0 10.0 30.0 50.0 70.0 90.0 110.0 130.0 150.0 170.0 190.0 210.0
12.0 11.0 10.0 9.0 8.0 7.0 6.0 5.0 4.0 3.0 2.0 1.0 0 -1.0 -2.0
X : parts per Million : Proton
1.0 2.0 3.0
(thousandths)
pestasulfamide B (2)

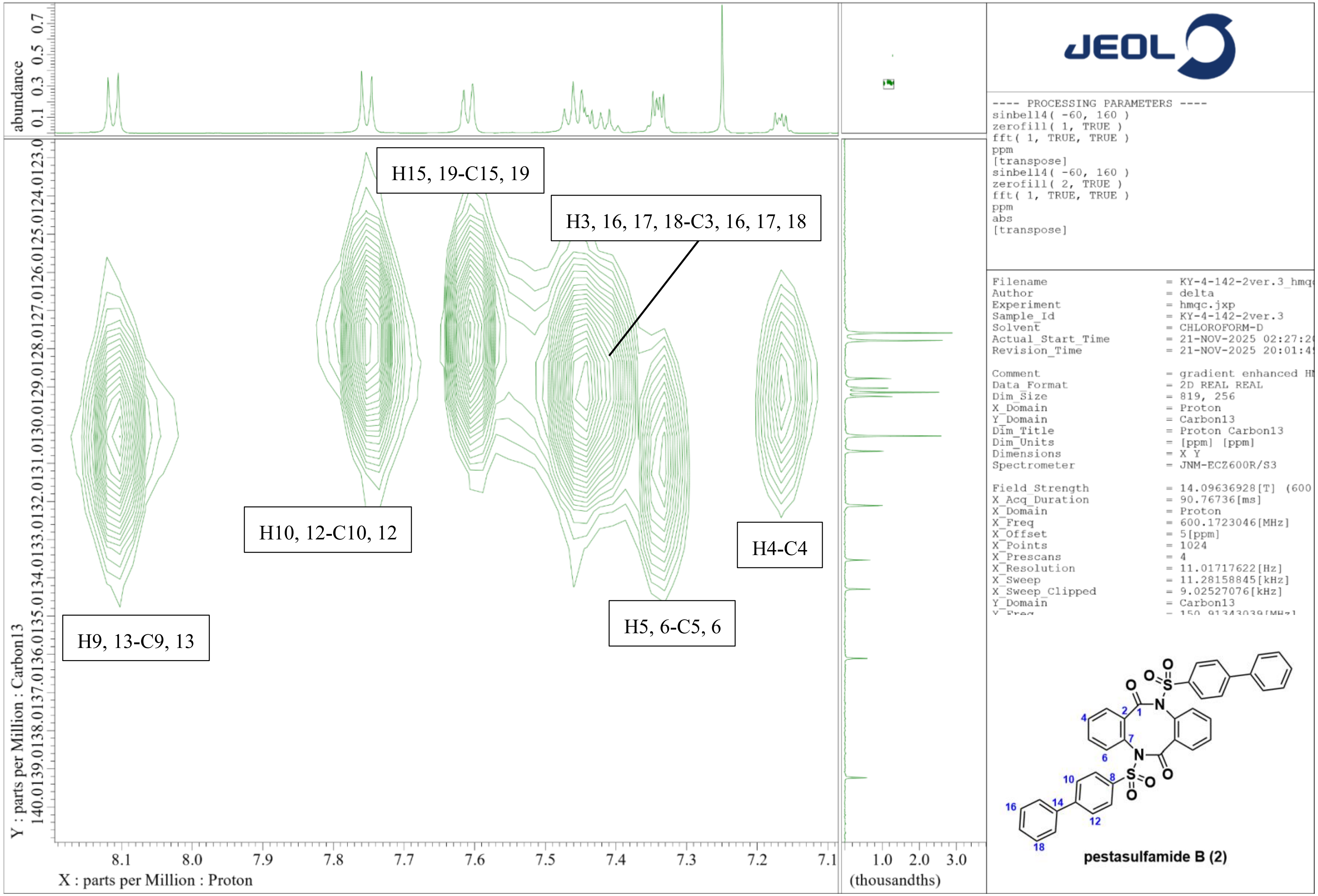

abundance
0.1 0.3 0.5 0.7
H15, 19-C15, 19
H3, 16, 17, 18-C3, 16, 17, 18
H10, 12-C10, 12
H4-C4
H5, 6-C5, 6
H9, 13-C9, 13
Y : parts per Million : Carbon13
140.0139.0138.0137.0136.0135.0134.0133.0132.0131.0130.0129.0128.0127.0126.0125.0124.0123.0
8.1 8.0 7.9 7.8 7.7 7.6 7.5 7.4 7.3 7.2 7.1
X : parts per Million : Proton
1.0 2.0 3.0
(thousandths)
JEOL
---- PROCESSING PARAMETERS ----
sinbell4(-60, 160)
zerofill(1, TRUE)
fft(1, TRUE, TRUE)
ppm
[transpose]
sinbell4(-60, 160)
zerofill(2, TRUE)
fft(1, TRUE, TRUE)
ppm
abs
[transpose]
Filename = KY-4-142-2ver.3_hmq
Author = delta
Experiment = hmqc.jxp
Sample_Id = KY-4-142-2ver.3
Solvent = CHLOROFORM-D
Actual_Start_Time = 21-NOV-2025 02:27:2
Revision_Time = 21-NOV-2025 20:01:4
Comment = gradient enhanced H
Data_Format = 2D REAL REAL
Dim_Size = 819, 256
X_Domain = Proton
Y_Domain = Carbon13
Dim_Title = Proton Carbon13
Dim_Units = [ppm] [ppm]
Dimensions = X Y
Spectrometer = JNM-ECZ600R/S3
Field_Strength = 14.09636928[T] (600
X_Acq_Duration = 90.76736[ms]
X_Domain = Proton
X_Freq = 600.1723046[MHz]
X_Offset = 5[ppm]
X_Points = 1024
X_Prescans = 4
X_Resolution = 11.01717622[Hz]
X_Sweep = 11.28158845[kHz]
X_Sweep_Clipped = 9.02527076[kHz]
Y_Domain = Carbon13
pestasulfamide B (2)

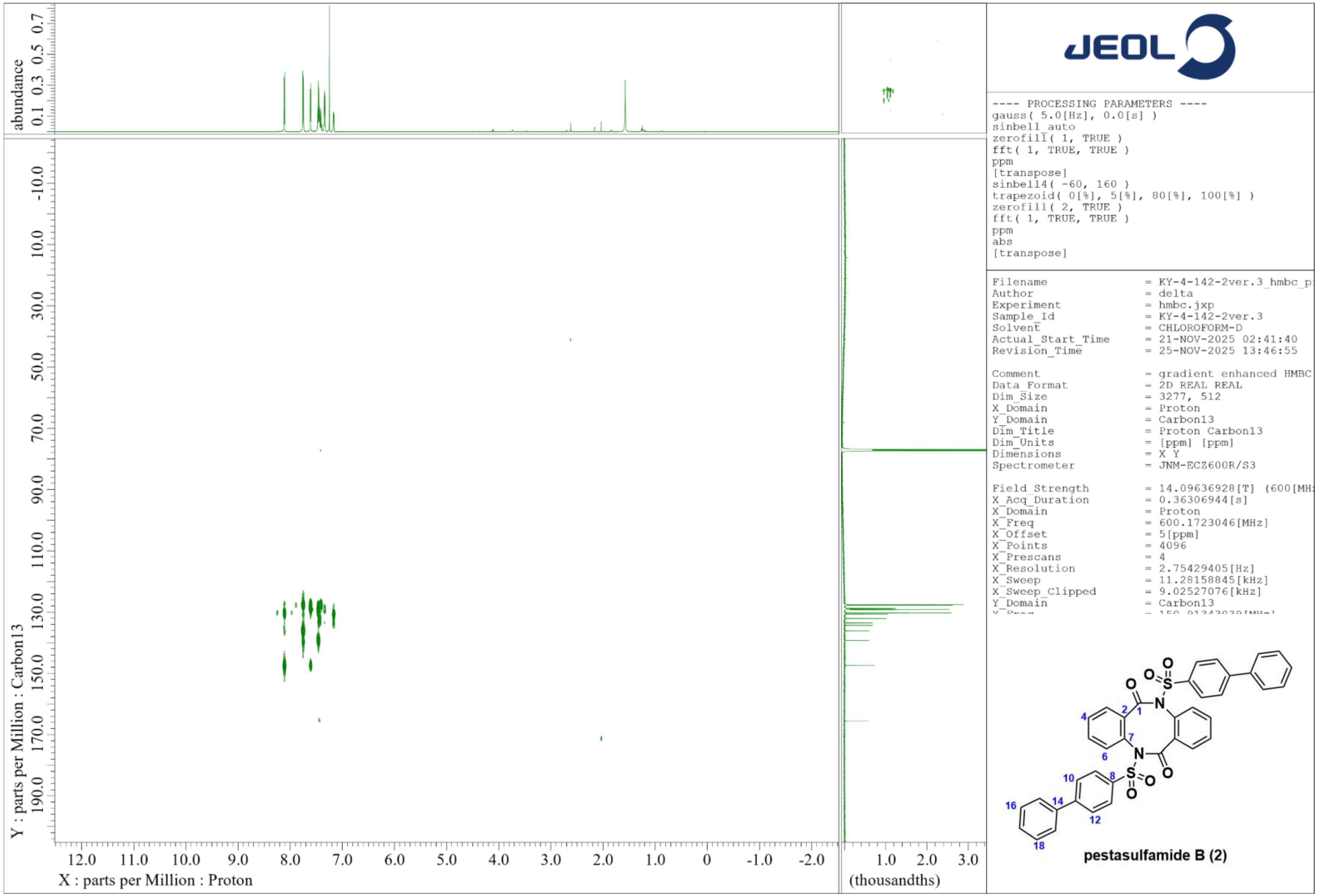
abundance
0.1 0.3 0.5 0.7
Y : parts per Million : Carbon13
-10.0 10.0 30.0 50.0 70.0 90.0 110.0 130.0 150.0 170.0 190.0
12.0 11.0 10.0 9.0 8.0 7.0 6.0 5.0 4.0 3.0 2.0 1.0 0 -1.0 -2.0
X : parts per Million : Proton
1.0 2.0 3.0
(thousandths)
---- PROCESSING PARAMETERS ----
gauss(5.0[Hz], 0.0[s])
sinbell_auto
zerofill(1, TRUE)
fft(1, TRUE, TRUE)
ppm
[transpose]
sinbell4(-60, 160)
trapezoid(0[%], 5[%], 80[%], 100[%])
zerofill(2, TRUE)
fft(1, TRUE, TRUE)
ppm
abs
[transpose]
Filename = KY-4-142-2ver.3_hmbc_p
Author = delta
Experiment = hmbc.jxp
Sample_Id = KY-4-142-2ver.3
Solvent = CHLOROFORM-D
Actual_Start_Time = 21-NOV-2025 02:41:40
Revision_Time = 25-NOV-2025 13:46:55
Comment = gradient enhanced HMBC
Data_Format = 2D REAL REAL
Dim_Size = 3277, 512
X_Domain = Proton
Y_Domain = Carbon13
Dim_Title = Proton Carbon13
Dim_Units = [ppm] [ppm]
Dimensions = X Y
Spectrometer = JNM-ECZ600R/S3
Field_Strength = 14.09636928[T] (600[MH
X_Acq_Duration = 0.36306944[s]
X_Domain = Proton
X_Freq = 600.1723046[MHz]
X_Offset = 5[ppm]
X_Points = 4096
X_Prescans = 4
X_Resolution = 2.75429405[Hz]
X_Sweep = 11.28158845[kHz]
X_Sweep_Clipped = 9.02527076[kHz]
Y_Domain = Carbon13
pestasulfamide B (2)

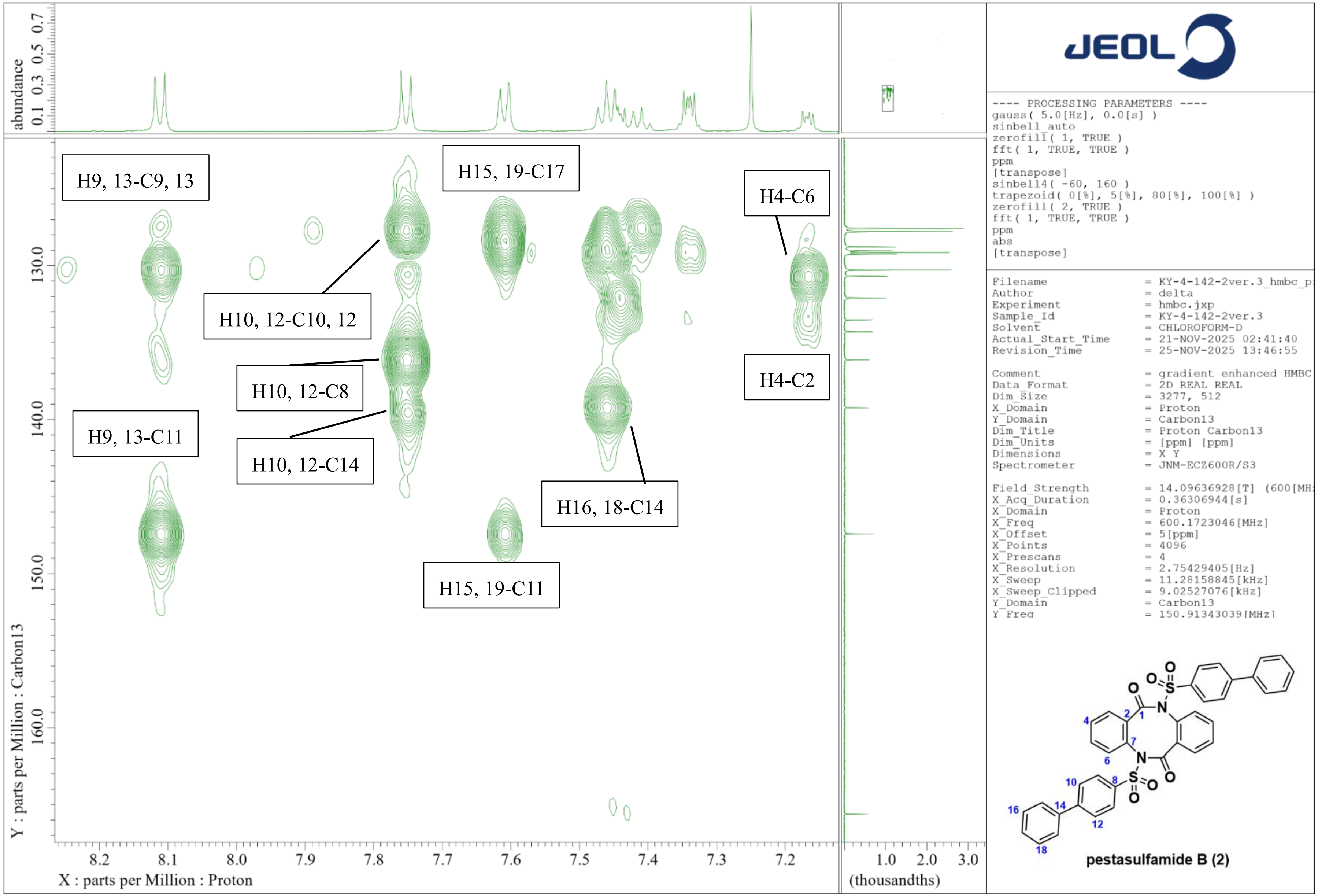
abundance
0.1 0.3 0.5 0.7
H9, 13-C9, 13
H15, 19-C17
H4-C6
H10, 12-C10, 12
H10, 12-C8
H4-C2
H9, 13-C11
H10, 12-C14
H16, 18-C14
H15, 19-C11
Y : parts per Million : Carbon13
130.0
140.0
150.0
160.0
8.2 8.1 8.0 7.9 7.8 7.7 7.6 7.5 7.4 7.3 7.2
X : parts per Million : Proton
1.0 2.0 3.0
(thousandths)
JEOL
---- PROCESSING PARAMETERS ----
gauss(5.0[Hz], 0.0[s])
sinbell_auto
zerofill(1, TRUE)
fft(1, TRUE, TRUE)
ppm
[transpose]
sinbell4(-60, 160)
trapezoid(0[%], 5[%], 80[%], 100[%])
zerofill(2, TRUE)
fft(1, TRUE, TRUE)
ppm
abs
[transpose]
Filename = KY-4-142-2ver.3_hmbc_p
Author = delta
Experiment = hmbc.jxp
Sample_Id = KY-4-142-2ver.3
Solvent = CHLOROFORM-D
Actual_Start_Time = 21-NOV-2025 02:41:40
Revision_Time = 25-NOV-2025 13:46:55
Comment = gradient enhanced HMBC
Data_Format = 2D REAL REAL
Dim_Size = 3277, 512
X_Domain = Proton
Y_Domain = Carbon13
Dim_Title = Proton Carbon13
Dim_Units = [ppm] [ppm]
Dimensions = X Y
Spectrometer = JNM-ECZ600R/S3
Field_Strength = 14.09636928[T] (600[MH
X_Acq_Duration = 0.36306944[s]
X_Domain = Proton
X_Freq = 600.1723046[MHz]
X_Offset = 5[ppm]
X_Points = 4096
X_Prescans = 4
X_Resolution = 2.75429405[Hz]
X_Sweep = 11.28158845[kHz]
X_Sweep_Clipped = 9.02527076[kHz]
Y_Domain = Carbon13
Y_Freq = 150.91343039[MHz]
pestasulfamide B (2)

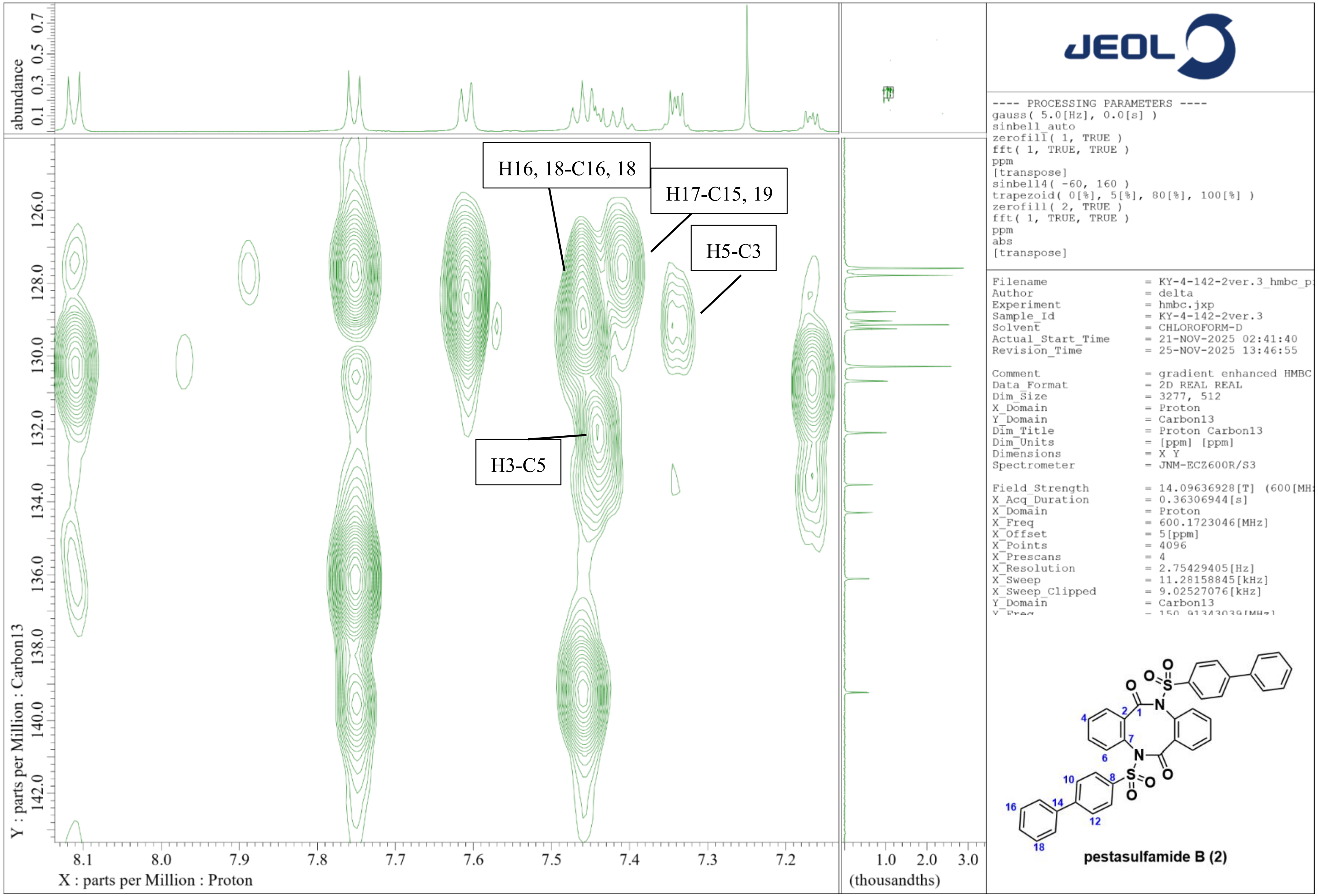
abundance
0.1 0.3 0.5 0.7
H16, 18-C16, 18
H17-C15, 19
H5-C3
H3-C5
Y : parts per Million : Carbon13
126.0 128.0 130.0 132.0 134.0 136.0 138.0 140.0 142.0
8.1 8.0 7.9 7.8 7.7 7.6 7.5 7.4 7.3 7.2
X : parts per Million : Proton
1.0 2.0 3.0
(thousandths)
JEOL
---- PROCESSING PARAMETERS ----
gauss(5.0[Hz], 0.0[s])
sinbell_auto
zerofill(1, TRUE)
fft(1, TRUE, TRUE)
ppm
[transpose]
sinbell4(-60, 160)
trapezoid(0[%], 5[%], 80[%], 100[%])
zerofill(2, TRUE)
fft(1, TRUE, TRUE)
ppm
abs
[transpose]
Filename = KY-4-142-2ver.3_hmbc_p
Author = delta
Experiment = hmbc.jxp
Sample_Id = KY-4-142-2ver.3
Solvent = CHLOROFORM-D
Actual_Start_Time = 21-NOV-2025 02:41:40
Revision_Time = 25-NOV-2025 13:46:55
Comment = gradient enhanced HMBC
Data_Format = 2D REAL REAL
Dim_Size = 3277, 512
X_Domain = Proton
Y_Domain = Carbon13
Dim_Title = Proton Carbon13
Dim_Units = [ppm] [ppm]
Dimensions = X Y
Spectrometer = JNM-ECZ600R/S3
Field_Strength = 14.09636928[T] (600[MH
X_Acq_Duration = 0.36306944[s]
X_Domain = Proton
X_Freq = 600.1723046[MHz]
X_Offset = 5[ppm]
X_Points = 4096
X_Prescans = 4
X_Resolution = 2.75429405[Hz]
X_Sweep = 11.28158845[kHz]
X_Sweep_Clipped = 9.02527076[kHz]
Y_Domain = Carbon13
pestasulfamide B (2)

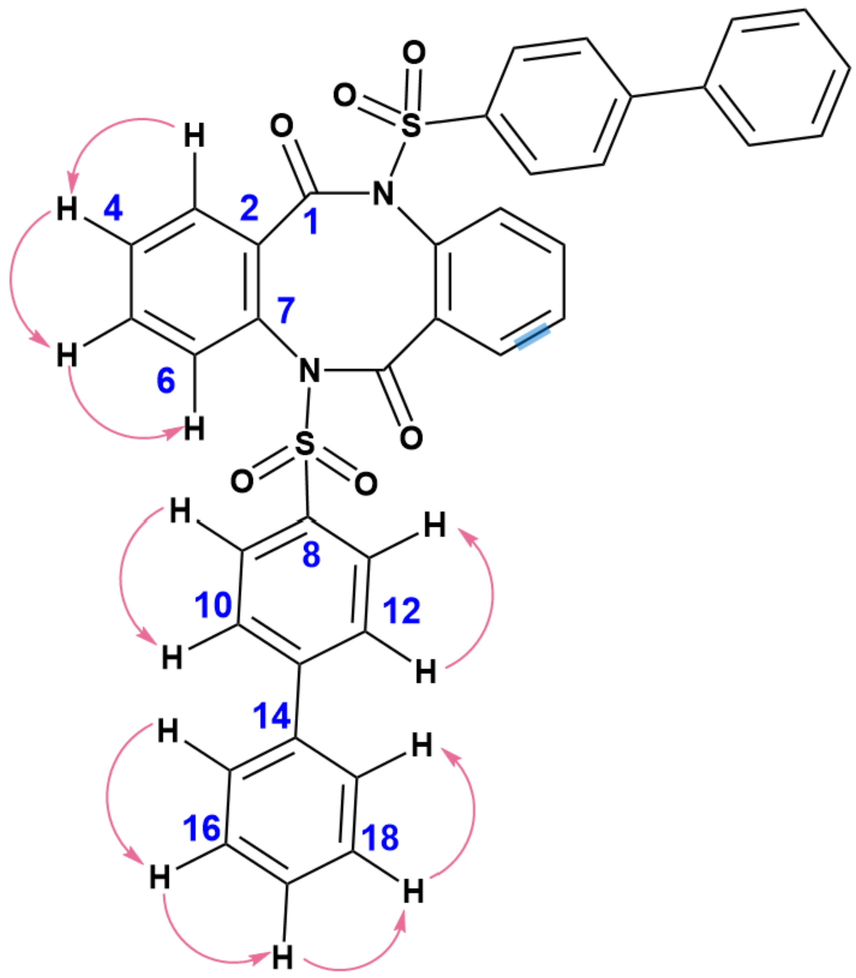

Figure S3. COSY correlations of pestasulfamide B (**2**).

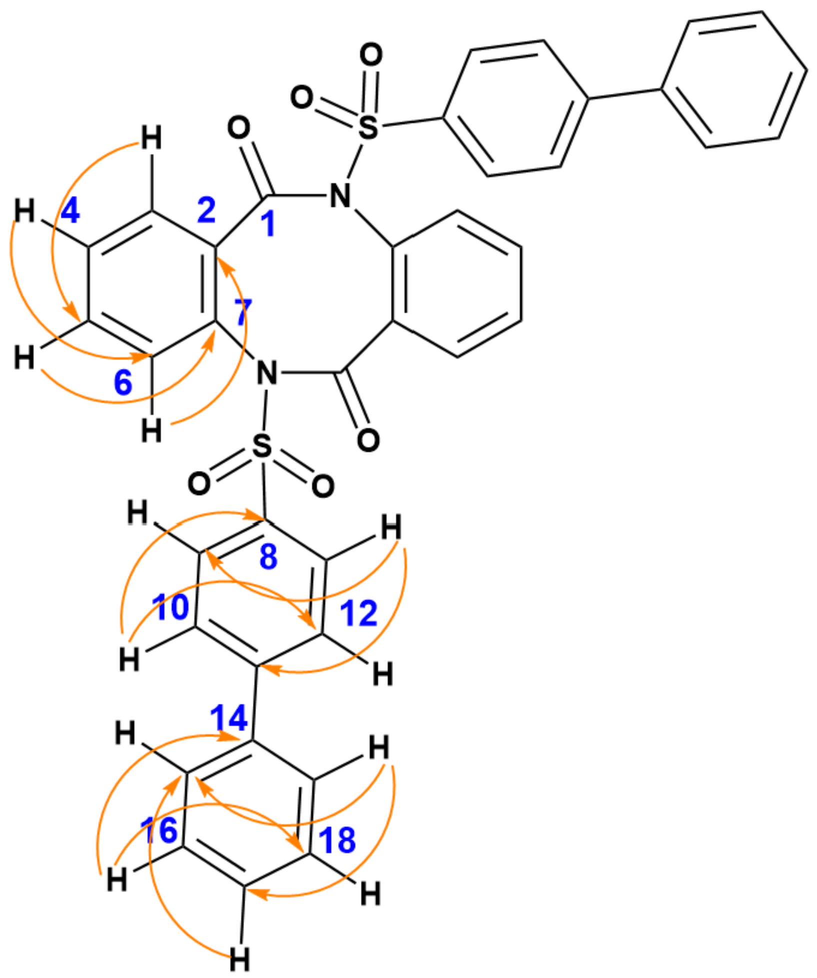

Figure S4. HMBC correlations of pestasulfamide B (**2**).

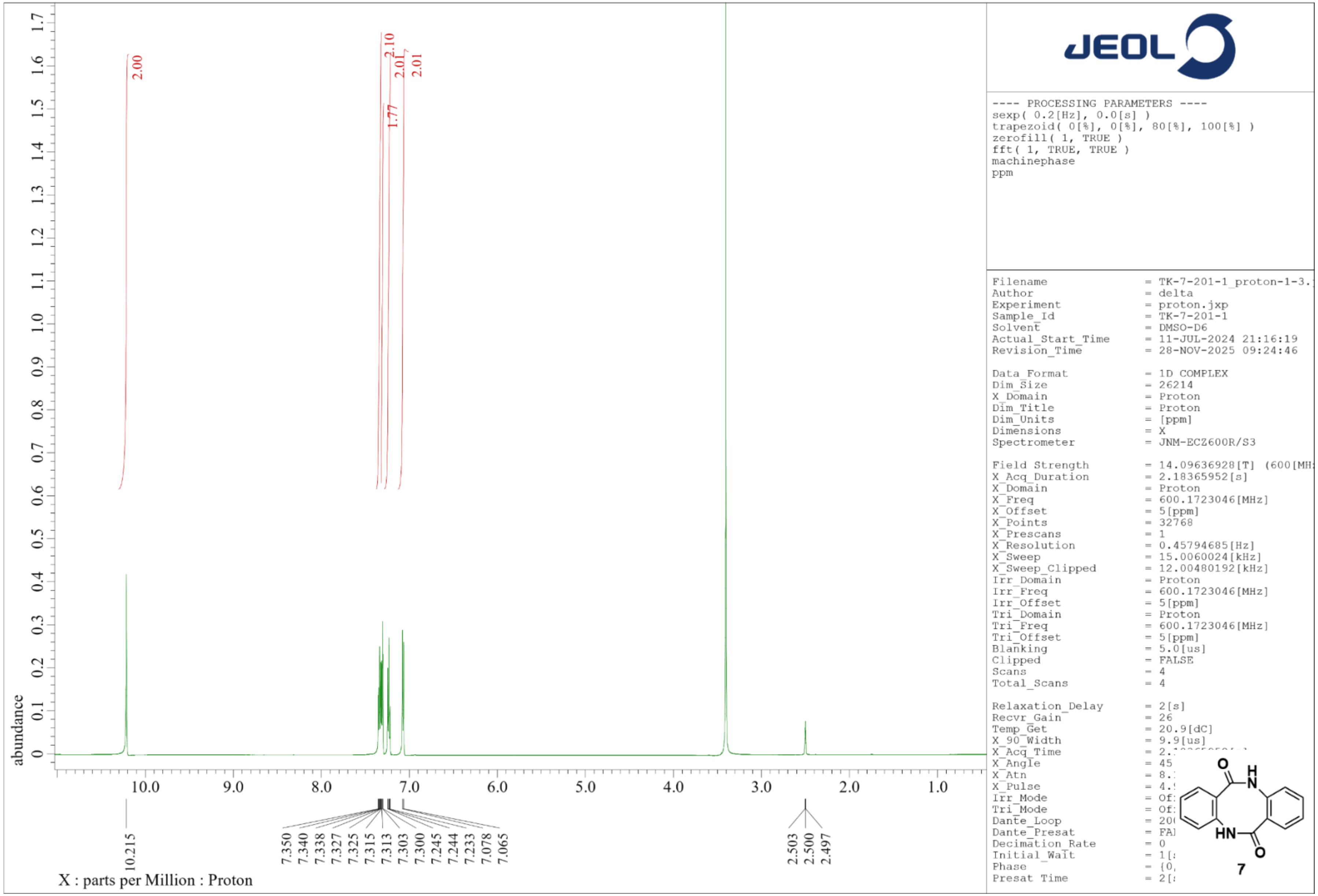
abundance
X : parts per Million : Proton
2.00
2.10
1.77
2.01
2.01
10.215
7.350
7.340
7.338
7.327
7.325
7.315
7.313
7.303
7.300
7.245
7.244
7.233
7.078
7.065
2.503
2.500
2.497
JEOL
---- PROCESSING PARAMETERS ----
sexp(0.2[Hz], 0.0[s])
trapezoid(0[%], 0[%], 80[%], 100[%])
zerofill(1, TRUE)
fft(1, TRUE, TRUE)
machinephase
ppm
Filename = TK-7-201-1_proton-1-3.
Author = delta
Experiment = proton.jxp
Sample_Id = TK-7-201-1
Solvent = DMSO-D6
Actual_Start_Time = 11-JUL-2024 21:16:19
Revision_Time = 28-NOV-2025 09:24:46
Data_Format = 1D COMPLEX
Dim_Size = 26214
X_Domain = Proton
Dim_Title = Proton
Dim_Units = [ppm]
Dimensions = X
Spectrometer = JNM-ECZ600R/S3
Field_Strength = 14.09636928[T] (600[MH
X_Acq_Duration = 2.18365952[s]
X_Domain = Proton
X_Freq = 600.1723046[MHz]
X_Offset = 5[ppm]
X_Points = 32768
X_Prescans = 1
X_Resolution = 0.45794685[Hz]
X_Sweep = 15.0060024[kHz]
X_Sweep_Clipped = 12.00480192[kHz]
Irr_Domain = Proton
Irr_Freq = 600.1723046[MHz]
Irr_Offset = 5[ppm]
Tri_Domain = Proton
Tri_Freq = 600.1723046[MHz]
Tri_Offset = 5[ppm]
Blanking = 5.0[us]
Clipped = FALSE
Scans = 4
Total_Scans = 4
Relaxation_Delay = 2[s]
Recvr_Gain = 26
Temp_Get = 20.9[dC]
X_90_Width = 9.9[us]
X_Acq_Time
X_Angle = 45
X_Atn
X_Pulse
Irr_Mode
Tri_Mode
Dante_Loop
Dante_Presat
Decimation_Rate = 0
Initial_Wait
Phase
Presat_Time
7

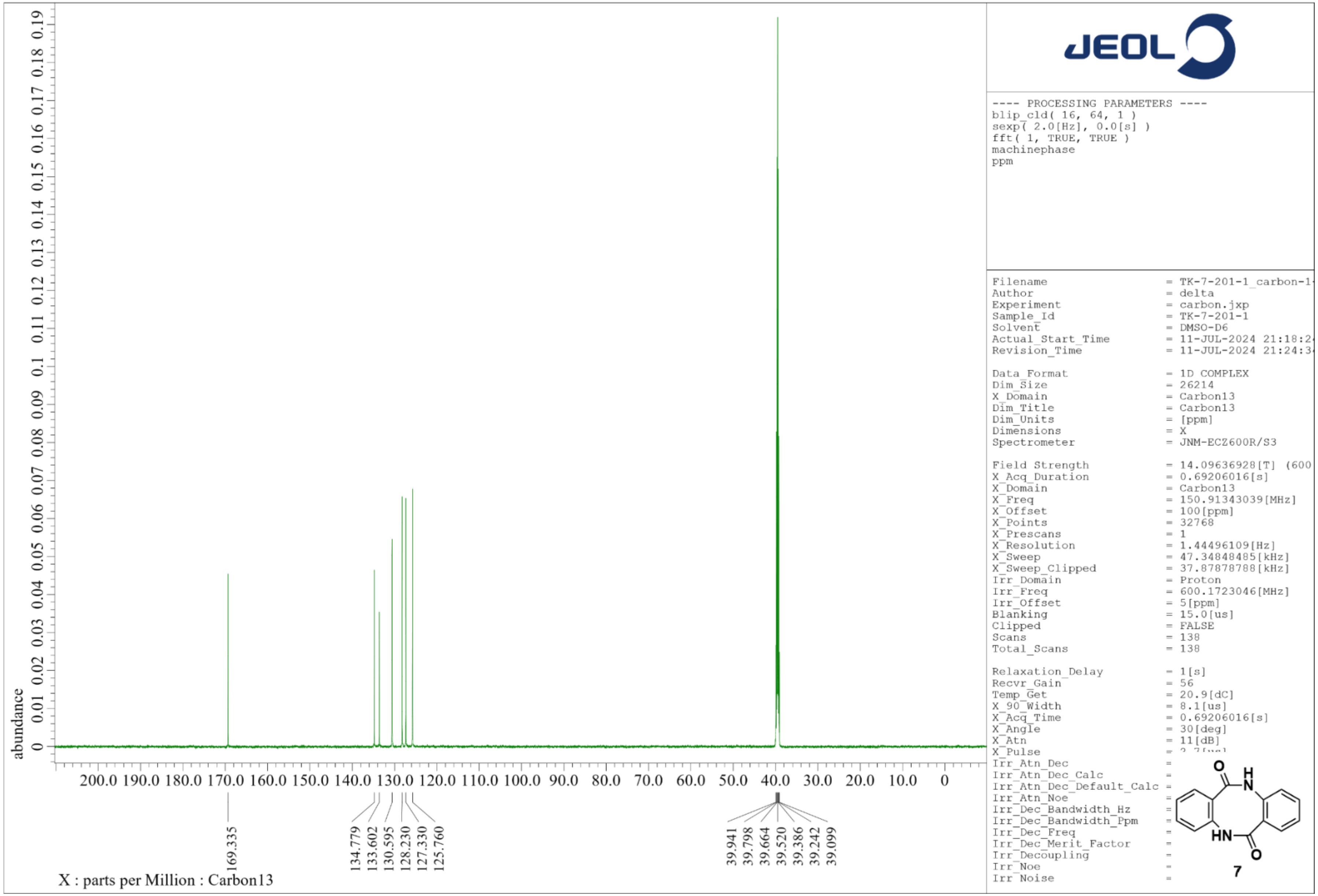
JEOL
---- PROCESSING PARAMETERS ----
blip_cld(16, 64, 1)
sexp(2.0[Hz], 0.0[s])
fft(1, TRUE, TRUE)
machinephase
ppm
Filename = TK-7-201-1_carbon-1
Author = delta
Experiment = carbon.jxp
Sample_Id = TK-7-201-1
Solvent = DMSO-D6
Actual_Start_Time = 11-JUL-2024 21:18:2
Revision_Time = 11-JUL-2024 21:24:3
Data_Format = 1D COMPLEX
Dim_Size = 26214
X_Domain = Carbon13
Dim_Title = Carbon13
Dim_Units = [ppm]
Dimensions = X
Spectrometer = JNM-ECZ600R/S3
Field_Strength = 14.09636928[T] (600
X_Acq_Duration = 0.69206016[s]
X_Domain = Carbon13
X_Freq = 150.91343039[MHz]
X_Offset = 100[ppm]
X_Points = 32768
X_Prescans = 1
X_Resolution = 1.44496109[Hz]
X_Sweep = 47.34848485[kHz]
X_Sweep_Clipped = 37.87878788[kHz]
Irr_Domain = Proton
Irr_Freq = 600.1723046[MHz]
Irr_Offset = 5[ppm]
Blanking = 15.0[us]
Clipped = FALSE
Scans = 138
Total_Scans = 138
Relaxation_Delay = 1[s]
Recvr_Gain = 56
Temp_Get = 20.9[dC]
X_90_Width = 8.1[us]
X_Acq_Time = 0.69206016[s]
X_Angle = 30[deg]
X_Atn = 11[dB]
X_Pulse =
Irr_Atn_Dec =
Irr_Atn_Dec_Calc =
Irr_Atn_Dec_Default_Calc =
Irr_Atn_Noe =
Irr_Dec_Bandwidth_Hz =
Irr_Dec_Bandwidth_Ppm =
Irr_Dec_Freq =
Irr_Dec_Merit_Factor =
Irr_Decoupling =
Irr_Noe =
Irr_Noise =
abundance
0 0.01 0.02 0.03 0.04 0.05 0.06 0.07 0.08 0.09 0.1 0.11 0.12 0.13 0.14 0.15 0.16 0.17 0.18 0.19
200.0 190.0 180.0 170.0 160.0 150.0 140.0 130.0 120.0 110.0 100.0 90.0 80.0 70.0 60.0 50.0 40.0 30.0 20.0 10.0 0
169.335
134.779
133.602
130.595
128.230
127.330
125.760
39.941
39.798
39.664
39.520
39.386
39.242
39.099
X : parts per Million : Carbon13
O
H
N
HN
O
7

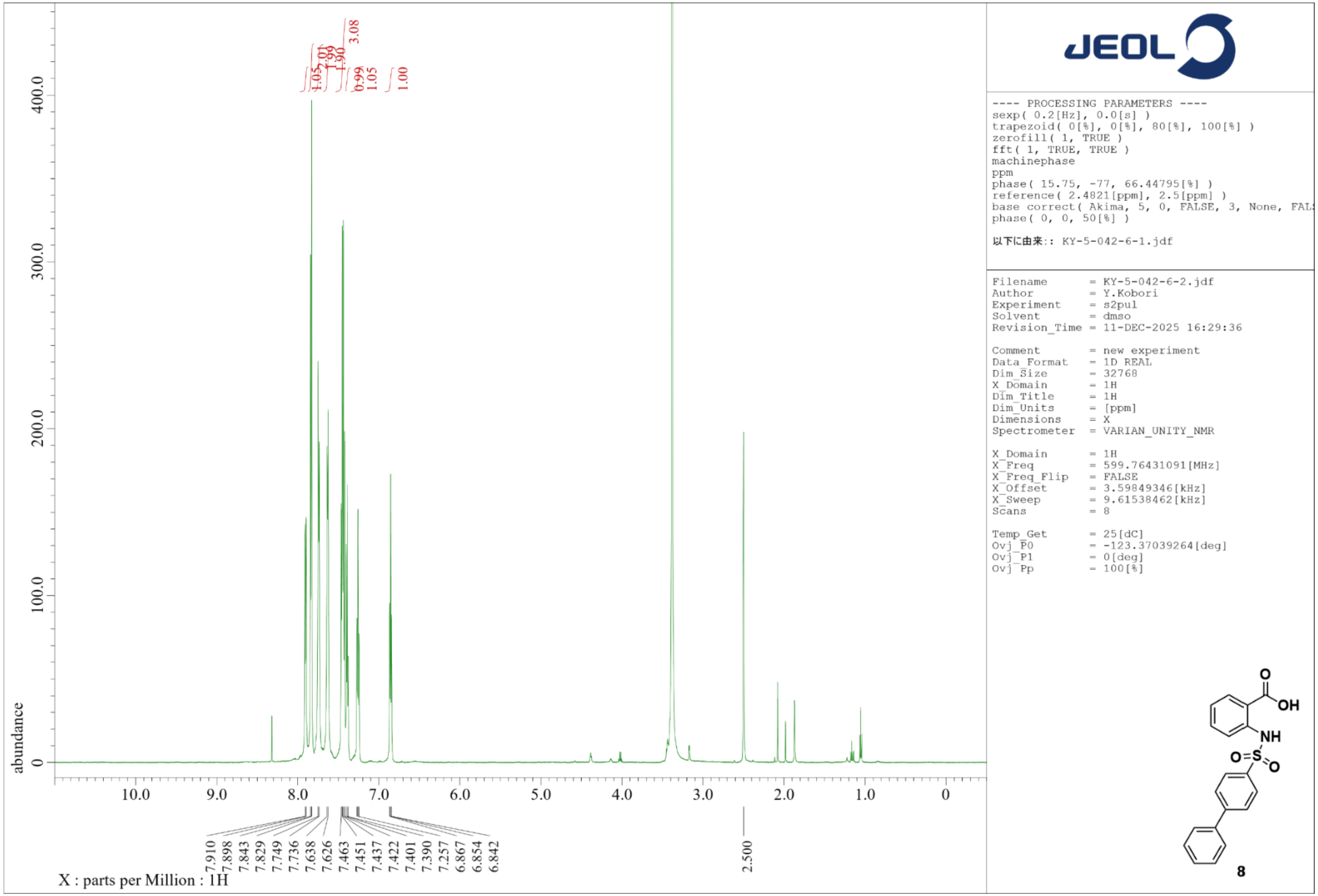
JEOL
---- PROCESSING PARAMETERS ----
sexp(0.2[Hz], 0.0[s])
trapezoid(0[%], 0[%], 80[%], 100[%])
zerofill(1, TRUE)
fft(1, TRUE, TRUE)
machinephase
ppm
phase(15.75, -77, 66.44795[%])
reference(2.4821[ppm], 2.5[ppm])
base correct(Akima, 5, 0, FALSE, 3, None, FAL
phase(0, 0, 50[%])
以下に由来:: KY-5-042-6-1.jdf
Filename = KY-5-042-6-2.jdf
Author = Y.Kobori
Experiment = s2pul
Solvent = dmso
Revision_Time = 11-DEC-2025 16:29:36
Comment = new experiment
Data_Format = 1D REAL
Dim_Size = 32768
X_Domain = 1H
Dim_Title = 1H
Dim_Units = [ppm]
Dimensions = X
Spectrometer = VARIAN_UNITY_NMR
X_Domain = 1H
X_Freq = 599.76431091[MHz]
X_Freq_Flip = FALSE
X_Offset = 3.59849346[kHz]
X_Sweep = 9.61538462[kHz]
Scans = 8
Temp_Get = 25[dC]
Ovj_P0 = -123.37039264[deg]
Ovj_P1 = 0[deg]
Ovj_Pp = 100[%]
abundance
0
100.0
200.0
300.0
400.0
1.05
1.01
1.99
1.90
3.08
0.99
1.05
1.00
10.0
9.0
8.0
7.0
6.0
5.0
4.0
3.0
2.0
1.0
0
7.910
7.898
7.843
7.829
7.749
7.736
7.638
7.626
7.463
7.451
7.437
7.422
7.401
7.390
7.257
6.867
6.854
6.842
2.500
X : parts per Million : 1H
O
OH
NH
O
S
O
8

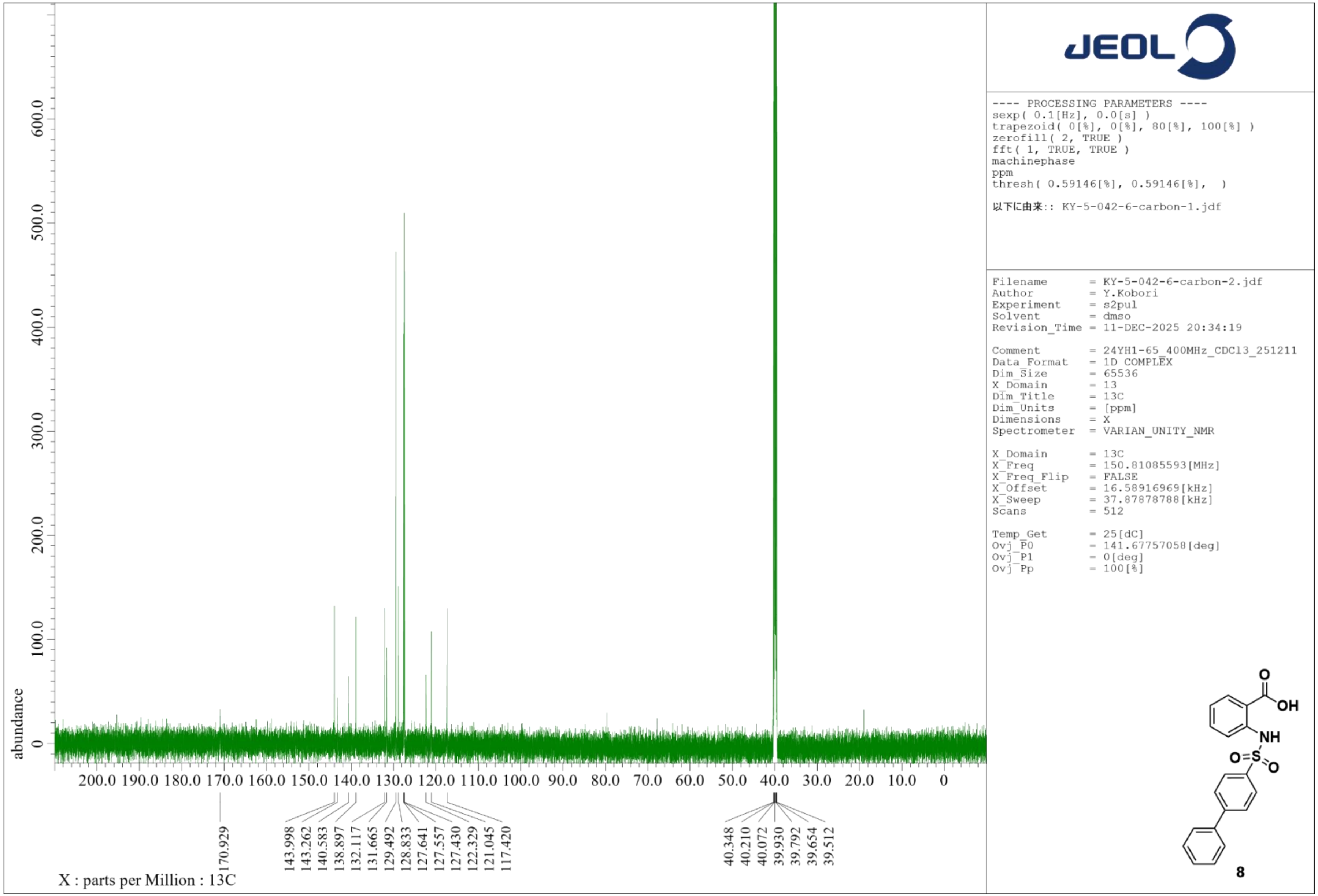
JEOL
---- PROCESSING PARAMETERS ----
sexp(0.1[Hz], 0.0[s])
trapezoid(0[%], 0[%], 80[%], 100[%])
zerofill(2, TRUE)
fft(1, TRUE, TRUE)
machinephase
ppm
thresh(0.59146[%], 0.59146[%],)
以下に由来:: KY-5-042-6-carbon-1.jdf
Filename = KY-5-042-6-carbon-2.jdf
Author = Y.Kobori
Experiment = s2pul
Solvent = dmso
Revision_Time = 11-DEC-2025 20:34:19
Comment = 24YH1-65_400MHz_CDCl3_251211
Data_Format = 1D COMPLEX
Dim_Size = 65536
X_Domain = 13
Dim_Title = 13C
Dim_Units = [ppm]
Dimensions = X
Spectrometer = VARIAN_UNITY_NMR
X_Domain = 13C
X_Freq = 150.81085593[MHz]
X_Freq_Flip = FALSE
X_Offset = 16.58916969[kHz]
X_Sweep = 37.87878788[kHz]
Scans = 512
Temp_Get = 25[dC]
Ovj_P0 = 141.67757058[deg]
Ovj_P1 = 0[deg]
Ovj_Pp = 100[%]
abundance
0
100.0
200.0
300.0
400.0
500.0
600.0
200.0 190.0 180.0 170.0 160.0 150.0 140.0 130.0 120.0 110.0 100.0 90.0 80.0 70.0 60.0 50.0 40.0 30.0 20.0 10.0 0
170.929
143.998
143.262
140.583
138.897
132.117
131.665
129.492
128.833
127.641
127.557
127.430
122.329
121.045
117.420
40.348
40.210
40.072
39.930
39.792
39.654
39.512
X : parts per Million : 13C
O
OH
NH
O=S=O
8
